# Multivariate Analysis of Cellular Uptake Characteristics for a (Co)polymer Particle Library

**DOI:** 10.1021/acsbiomaterials.3c01803

**Published:** 2024-02-20

**Authors:** Stefan Baudis, Toralf Roch, Maria Balk, Christian Wischke, Andreas Lendlein, Marc Behl

**Affiliations:** † Institute of Active Polymers, Helmholtz-Zentrum Hereon, Kantstraße 55, 14513 Teltow, Germany; ‡ Institute of Biochemistry and Biology, University of Potsdam, Karl-Liebknecht-Str. 24-25, 14476 Potsdam-Golm, Germany

**Keywords:** copolymer library, automated synthesis, principal component analysis, cellular uptake, nanoparticle

## Abstract

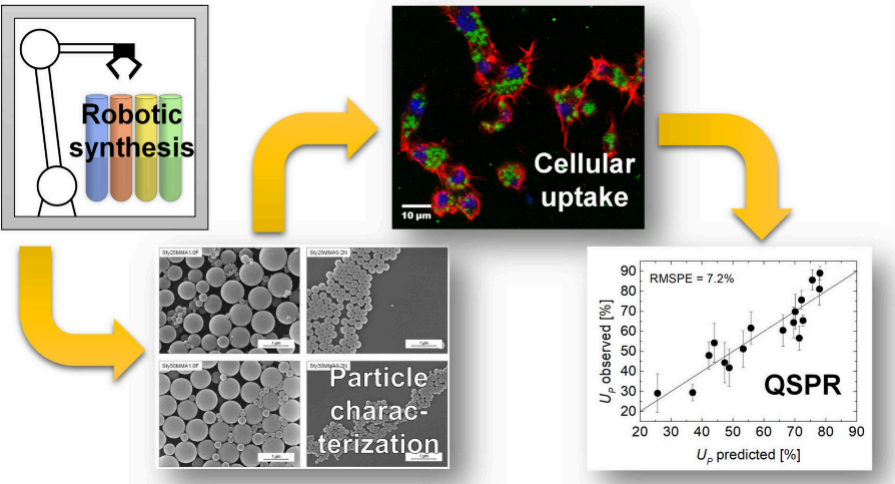

Controlling cellular responses to nanoparticles so far is predominantly empirical, typically requiring multiple rounds of optimization of particulate carriers. In this study, a systematic model-assisted approach should lead to the identification of key parameters that account for particle properties and their cellular recognition. A copolymer particle library was synthesized by a combinatorial approach in soap free emulsion copolymerization of styrene and methyl methacrylate, leading to a broad compositional as well as constitutional spectrum. The proposed structure–property relationships could be elucidated by multivariate analysis of the obtained experimental data, including physicochemical characteristics such as molar composition, molecular weight, particle diameter, and particle charge as well as the cellular uptake pattern of nanoparticles. It was found that the main contributors for particle size were the polymers’ molecular weight and the zeta potential, while particle uptake is mainly directed by the particles’ composition. This knowledge and the reported model-assisted procedure to identify relevant parameters affecting particle engulfment of particulate carriers by nonphagocytic and phagocytic cells can be of high relevance for the rational design of pharmaceutical nanocarriers and assessment of biodistribution and nanotoxicity, respectively.

## 1 Introduction

Particle-based therapeutics^1^ is a diverse group of carriers, which are promising means for different disease scenarios. These systems range, e.g., from colloidal associates like micelles, liposomes, or extracellular vesicles^2^ to lipid^3^ and polymer particles of different sizes.^4,5^ The unique properties of particulate systems in the submicro scale hold promise for their accumulation in the tissue of interest via specific interactions.^6,7^ This, especially compared to large scale devices, includes injectability and–at least conceptually–the ability for biodistribution within the body with the bloodstream. For instance, the particle-induced engulfment of otherwise not as efficiently recognized molecules by immune cells was established several years ago.^8,9^ This has come to the awareness of a broad community along with the recent rollout of mRNA vaccines.^10−12^

A proper design of polymeric nanoparticles can be considered a key element in matching applicational needs. Besides particle size^13^ and shape,^14^ also other characteristics of the polymer matrix and the particles’ surface properties must not be neglected.^15,16^ Noteworthy, the practical relevance of the particle shape for cellular uptake is controversial in the case of multiple cell-particle contacts.^17,18^ There has been a trend to decorate nanocarriers at their surfaces with individual or combinations of targeting molecules^19^ or cell membrane motifs.^20^ This is reasonable when considering that the recognition of particles in a biosystem occurs primarily through molecules at their surfaces. In this respect, a high structural complexity of carriers, which might further increase through in vivo modifications (protein adsorption and/or ligand degradation in biofluids^21^) can set hurdles in terms of the applicable regulatory framework and the reproducible quality of nanocarriers. While ligand coverage of nanoparticles has its value in advanced research on drug targeting, it is still of interest to understand the effect of the chemical composition of matrix polymers on particle properties and cellular recognition.^22^ For instance, there is evidence that a series of copolymer particles can be recognized and endocytosed by human cells substantially different based on the polymers’ comonomer ratio.^23^ In order to more efficiently streamline the design of particulate carriers, the experimental observations (e.g., on particle characteristics) can be supported by *in silico* methodologies of various ranges of complexity and reliability especially if available data sets are limited.^24^

To efficiently evaluate structure–property-biorecognition relationships, combinatorial screening has been identified as a suitable approach that can be conducted in various modes (single particle-type screening; pooled particle screening).^25^ A key enabling technology is the provision of polymer libraries, which are accessible by high throughput synthesis and characterization, as summarized in a recent review.^26^ On the one hand, for preparing particle libraries, the processes of (i) polymer synthesis and (ii) particle formation can be separated from each other. In this context, a very prominent technique for the preparation of tailor-made particles (including whole spheres and core–shell constructs) is microfluidics.^27^ The conventional procedure is that polymer or precursor solutions are pumped through microfluidic devices, and particles are formed^28^ and fixated by fast (cross-linking) reactions,^29^ typically by ionic interactions, thermal or photopolymerization.^30^ Alternative techniques for particle formation include spraying methods (e.g., by surface acoustic wave^31^ or electrospraying^32^) and Layer-by-Layer assembly.^33^ These techniques are able to set particle and composition parameters independently, which not only can be an advantage but also drastically reduces throughput. A higher throughput for such approaches may be attained by parallelization.^34^ Emulsion polymerization, on the other hand, allows an integrated process of polymer synthesis and particle generation. The establishment of such complex synthetic procedures for robotic synthesizer platforms opened up the possibility for a wide screening of such material libraries.^35,36^

For a design of particles that could fulfill specific tasks in biology, central considerations are particle-cell interactions, especially in cellular uptake processes.^37−39^ Cellular uptake is driven by different factors, determined by the nature of the particles as well as the cell type.^37,39^ Some of the most important particle-originated factors are size, shape, and (surface) chemistry,^40^ with the effective pK_A_ being additionally relevant when employing charged polymers for complexation.^41^ In order to elucidate these processes, methyl methacrylate has before been copolymerized with anionic or cationic comonomers that had a dominating effect on particle size, surface texture,^42^ and endocytosis.^43^ Here, we chose a copolymer system without free charged groups, methyl methacrylate (MMA) combined with styrene (Sty), very commonly used as cell cultured materials in its polymeric form^44^ to produce a gradient in hydrophobicity as shown for polymer brushes,^45^ composites,^46^ membranes,^47^ and cell substrates.^48^ A broad compositional (copolymer ratio) as well as constitutional (particle size, zeta potential) spectrum was obtained, which enabled the elucidation of the cellular uptake characteristics for two different cell types (phagocytic and nonphagocytic) by a multivariate analysis of the obtained experimental data.

Similar previous studies to decipher structure-property relationships for cellular uptake of particulates^49^ emphasized single parameter, most importantly the particle size,^50−52^ the particle shape,^53−55^ the surface topology and charge,^51,56^ and hydrophobicity,^56^ very often in complex material constructs or even by lithographic or 3D printing methods.^57,58^ Here, we intentionally chose this very simple system to enable a bottom-up consideration of the final biological effects from the constitution of single particles from single polymer chains in a multivariate mode, with the goal to provide a methodological blueprint to be applied to other materials to identify specific parameters accounting for uptake in these materials.

## 2 Materials and Methods

### 2.1 Materials

Monomers were purchased from Sigma-Aldrich (Steinheim, Germany). Methyl methacrylate (MMA, ≥99%, Fluka) was freshly distilled prior use. Styrene (Sty, ≥99%) was extracted three times with a 10% aqueous sodium hydroxide solution, dried over anhydrous magnesium sulfate, and distilled *in vacuo* (and stored at −20 °C unless not polymerized immediately). Fluorescein-*O*-acrylate (FA, 95%) was used as a solution in ethanol (quality for molecular biology) in a concentration of 4 mg/mL. Ammonium persulfate (APS, >98%, Merck, Darmstadt, Germany) was recrystallized from an ethanol/water mixture and used as an aqueous solution with a concentration of 34 mg/mL. Ammonium bicarbonate (ABC, >99%, Bernd Kraft, Duisburg, Germany) was used as an aqueous solution with a concentration of 71 mg/mL. Water used to prepare the solutions and as a reaction- and washing medium was of “water for injection” quality (WFI) (Thermo Scientific HyClone HyPure WFI; provider: Fisher Scientific, Schwerte, Germany).

### 2.2 High Throughput Synthesis and Characterization of the Polymer Library

High throughput (HT) synthesis was performed by parallel polymerization reactions in three reactor arrays with 16 reactors each (with a round-bottom cylindrical shape, an inner diameter of ∼16 mm, effective volume of 7.5 mL) employing the automated parallel synthesizer platform Accelerator SLTII/106 (Chemspeed Technologies, Augst, Switzerland). Lines of the robotic volumetric transfer system were consecutively rinsed with a 1 M aqueous sodium hydroxide solution, 1 M hydrochloric acid, and ethanol before the system was filled with WFI. This procedure is proposed to minimize the endotoxin burden of the final products.^59^ The soap-free emulsion polymerization (also refer to Scheme S1 in the Supporting Information) of MMA and Sty in ABC buffered solutions was conducted using APS as the initiator by varying the absence/presence of fluorescence labeling using FA as the dye (in very low concentrations), the MMA/Sty molar ratios, and the monomer concentrations. For details on the synthesis, please refer to the Supporting Information (Section 1.2, Table S1, and Scheme S2). Purification of the polymer particles was performed by dialysis using the filter units of Corning Costar Spin-X polypropylene centrifuge tube filters with a 0.22 μm pore size nylon membrane purchased from Sigma-Aldrich (Steinheim, Germany). Further details can be found in the Supporting Information (Section 1.4). HT characterization (composition and molecular weight) was performed with particles directly obtained from the HT synthesis. FTIR spectra were recorded in DRIFT mode (Diffuse Reflectance Infrared Fourier Transform) using a Bruker Vertex 70 spectrometer (Bruker Optik, Ettlingen, Germany) with the HT extension HTS-XT as reported earlier.^36^ Further details can be found in the Supporting Information (Section 1.3). Molecular weights were determined with the HT gel permeation chromatography (GPC) system Tosoh EcoSEC HLC-8320 GPC (Tosoh Bioscience, Stuttgart, Germany) in THF using overlapped sample injection to increase the throughput.^35^ Further details can be found in the Supporting Information (Section 1.3).

### 2.3 Characterization of Particle Size, Charge, and Morphology

Particle sizes were determined by dynamic light scattering (DLS) of diluted latex particle samples in water at 25 °C in quartz glass cuvettes using a Beckman Coulter Delsa Nano C (Beckmann Coulter GmbH, Krefeld, Germany). DLS patterns were analyzed at an angle of 165° using the CONTIN fit. Particle size and morphology were additionally assessed by scanning electron microscopy (SEM) on a Carl Zeiss NTS Gemini SupraTM 40 VP (Carl Zeiss Microscopy GmbH, Oberkochen, Germany) at 1 kV with a secondary electron detector. Zeta potential of the particles was determined by laser doppler microelectrophoresis of diluted latex particle samples in water at 25 °C using a Malvern Zetasizer Nano ZS (Malvern Instruments GmbH, Herrenberg, Germany).

### 2.4 Cell Studies

For the detection of soluble endotoxins, the dialysates of all particles were analyzed by the LAL test (Lonza, Cologne, Germany), which was performed according to the manufacturer’s instructions.

Additionally, a cell-based endotoxin determination assay was performed as previously described.^60^ In brief, HEK-Blue-hTLR4 and HEK-Blue-Null2 RAW-Blue (InvivoGen, San Diego, USA) activation assays were performed with 5 × 10^5^ cells suspended in 200 μL of VLE-DMEM (Biochrom) and cultured in 96 well plates (Corning Costar, Sigma-Aldrich, Germany). The cells were exposed to different particle suspensions or lipopolysaccharides (LPS) from *E. coli* strain O111:B4 (Axxora GmbH, Lörrach, Germany) as a positive control for 24 h. After incubation, cell culture supernatants were harvested and analyzed for alkaline phosphatase activity using a QuantiBlue solution (Invivogen). The cell survival rate was quantified by flow cytometry using 1 μg·mL^–1^ 4,6-diamidino-2-phenylindole (DAPI), which was added immediately prior to analysis.

For uptake studies, the RAW264.7-Blue and HEK-Blue cells (both Invivogen) were labeled with the cell tracker Dye eFluor 670 (eBioscience, Frankfurt/Main, Germany) according to the manufacturer’s instructions to clearly discriminate cells from particles. The labeled cells were incubated for 24 h with increasing concentrations of the different fluorescence labeled particles. The theoretical values of particle concentrations as obtained from the particle synthesis were taken as a basis, which were corrected by the real values obtained from the solid content determinations (see section 2.3). Cells were harvested using Accutase (Life Technologies, Darmstadt, Germany), washed with autoMACS running buffer, and analyzed using a MACSQuant flow cytometer (both Miltenyi Biotec, Bergisch Gladbach, Germany). Thorough repeated washing of the cells applying a well-established protocol^18,23^ before the flow cytometric measurements ensured exclusion of all particles, which are just adhering to the cell surface. To discriminate live and dead cells, 1 μg·mL^–1^ DAPI was added to the cell suspensions immediately prior to cell acquisition. Flow cytometry data were analyzed using the FlowJo software v10 (Tree Star, Ashland, USA).

### 2.5 Data Analysis

Principal component analysis (PCA) and multiple linear regression (MLR) were performed with the Microsoft Excel Add-in Multibase (ver. 2015, provided by Numerical Dynamics). Data preparation comprised scaling by the standard deviations and mean centering for PCA. Unprocessed data were used for MLR. For the analysis of biological data and their graphical representation, GraphPad Prism v8.0 (La Jolla, CA 92037, USA) was used.

## 3 Results and Discussion

### 3.1 (Co)polymer Particle Library HT Synthesis and Characterization

**Figure 1:**
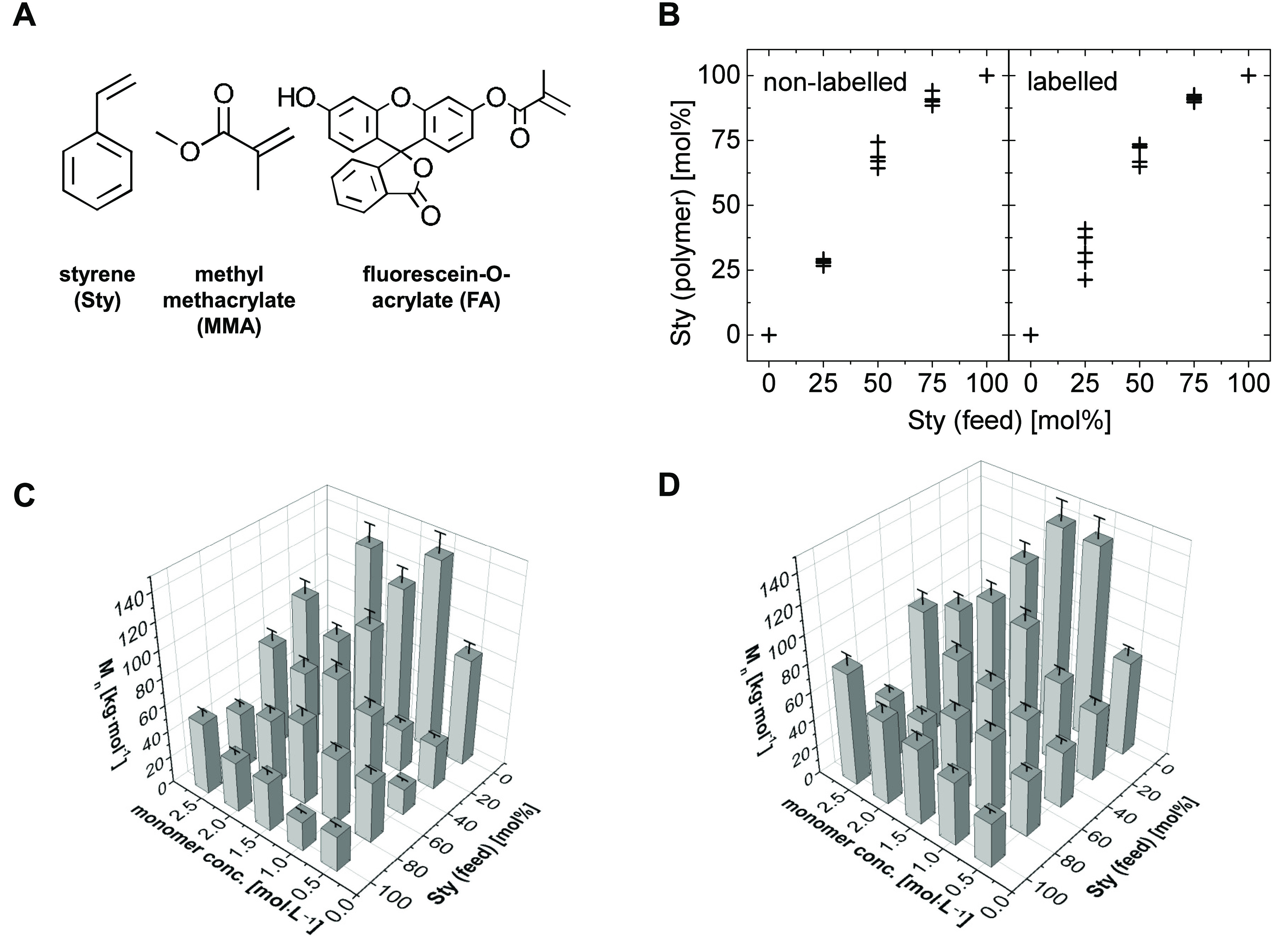
Results of the high throughput characterization of the particle library: (A) Molecular structure of the (co)monomers of the polymer library. (B) Particle composition of the whole library based on NIR data compared to feed ratios (data for a given feed include all samples synthesized at different monomer concentrations for the same monomer ratios). Error bars for the composition were omitted for clarity reasons (characteristic error ± 2 mol %^36^). (C) Molecular weight of nonlabeled polymer particles (GPC) and (D) molecular weight of fluorescence-labeled polymer particles (GPC). Error bars indicate the margin of error attributed to the error in molecular weight determination by GPC (±10%). Data set and 2D versions of the diagrams with the color code are provided in the Supporting Information.

A 48-membered combinatorial library of particles consisting of poly(methyl methacrylate-*co*-styrene) was synthesized by soap-free emulsion polymerization (schematic can be found in the Supporting Information, Scheme S1) via parallel synthesis employing a robotic synthesizer. In soap free emulsion polymerization, hydrophobic monomers with minimal aqueous solubility are emulsified in water. Given their limited solubility, monomers can diffuse in the water phase, where polymerization is initiated and growing polymer chains build micelles that further develop to become solid polymeric lattices.^61^ To avoid an induction period of the polymerization by inhibition by oxygen (dissolved in aqueous media), the synthesis procedure comprised a preinitiation step, in which the initiator APS was added, and the temperature was raised and cooled down again (oxygen quenching). Then, the monomers (styrene, Sty, methyl methacrylate, MMA, with or without fluorescein-*O*-acrylate, FA; Figure 1A) and more APS were added, and the emulsion polymerization was performed. Additionally, after the regular polymerization time, a postinitiation step was applied by adding another amount of APS to increase monomer conversion (a flowchart of the procedure is provided in the Supporting Information, Scheme S2). The particles of the combinatorial library varied in a comonomer ratio (five different molar ratios in the feed) and monomer concentration (up to five different concentrations in the reaction mixture, Table S1 in the Supporting Information). The copolymer particles were denoted as Sty[X]MMA[Y][Z], where [X] is the molar ratio of Sty [mol %] in the feed, [Y] is the concentration factor, and [Z] is the index for the labeling (“N” for nonlabeled and “F” for fluorescence-labeled particles), e.g. Sty25MMA0.8F was prepared by using a 25 mol % Sty feed at a monomer concentration of 2.10 mmol·mL^–1^ (80% of the highest concentration, i.e., concentration factor 0.8) including fluorescence labeling (see Table S1).

A high throughput characterization of the particles was performed. The analysis of copolymer particle composition based on NIR spectra^36^ suggested that the copolymerization was successful (Figure 1A). The composition of the polymeric particles followed the trend as expected for the comonomer system, namely, preferred incorporation of Sty into the copolymer (e.g., 70 mol % Sty in particles with a 50 mol % Sty feed), as should be a consequence of the reactivities, aqueous solubilities, diffusivities, and phase partition of the two monomers into the growing micelles. The standard deviation of the results for the composition of the fluorescence labeled particles was higher compared to the corresponding variance of the nonlabeled particles. This may be attributed to two effects: (i) slightly different conditions during the synthesis procedure (mainly the addition of ethanol as a solvent for the labeling agent fluorescein-*O*-acrylate) and (ii) a slight difference in infrared spectra by the present fluorescence dye as the method (multivariate calibration) for the determination of the composition based on NIR spectra was established with nonlabeled copolymers. By trend, the number-average of the molecular weight *M̅*_n_ as determined by GPC^35^ increased with increasing monomer concentration (or concentration factor, respectively), while it decreased with the molar ratio of Sty in the feed (Figure 1B and C, data set and 2D versions of the diagrams with the color code are provided in the Supporting Information, Table S2 and Figure S1). With an increasing molar content of Sty, an increasing hydrophobicity of the copolymers could be demonstrated as visible by increasing water contact angles (Figure S2) for thin films obtained by melt-compression of nanoparticles.

**Figure 2:**
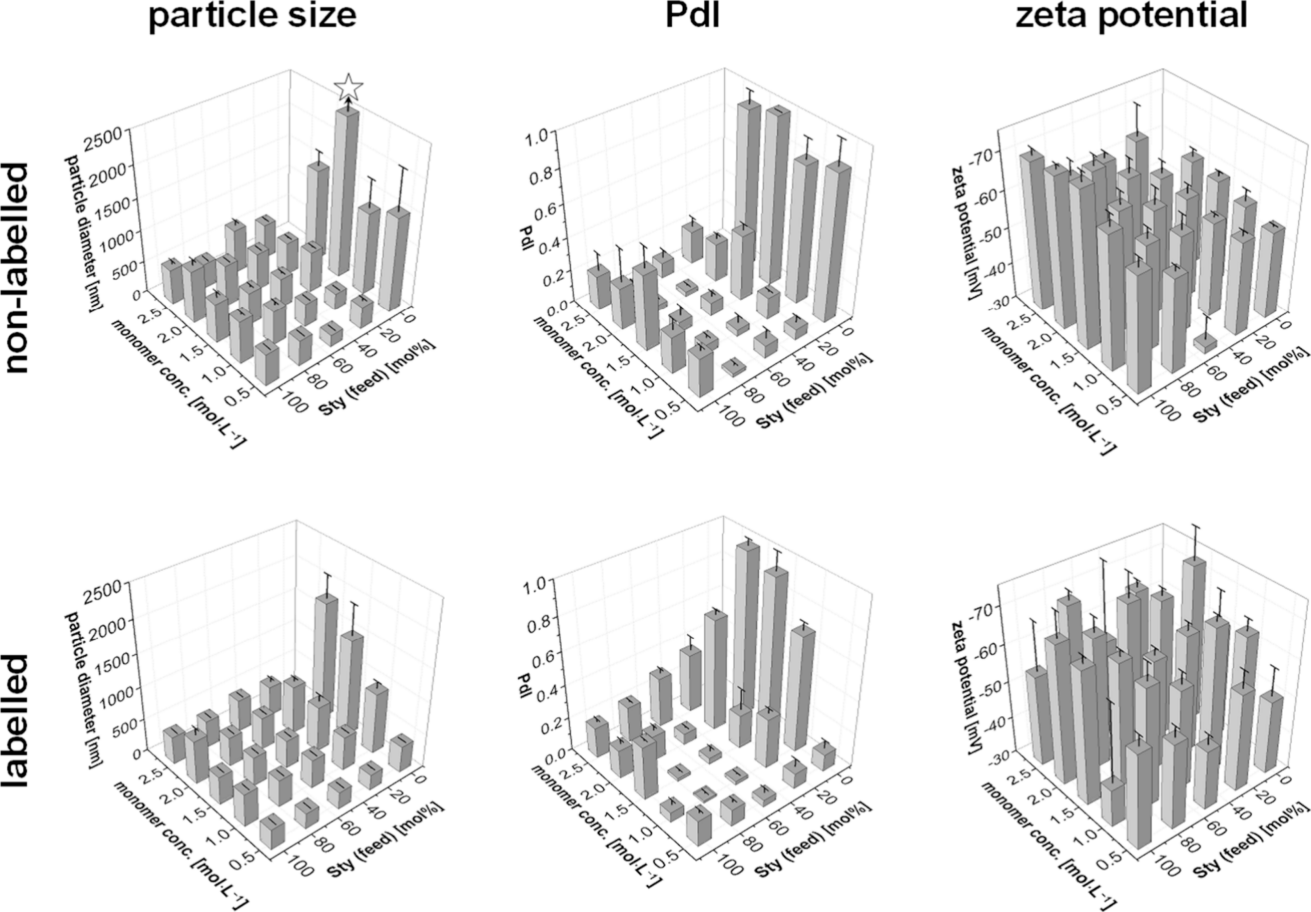
Particle sizes and polydispersity indices (PdIs) from DLS analysis as well as zeta potential, all for dialyzed particle suspensions, depending on the feed ratio of styrene (Sty) and the comonomer concentration. Error bars indicate the standard deviation of the measurements; the star on the diagram for the particle size of nonlabeled particles indicates large aggregates above 5000 nm. The data set and 2D versions of the diagrams with the color code are provided in the Supporting Information.

**Figure 3:**
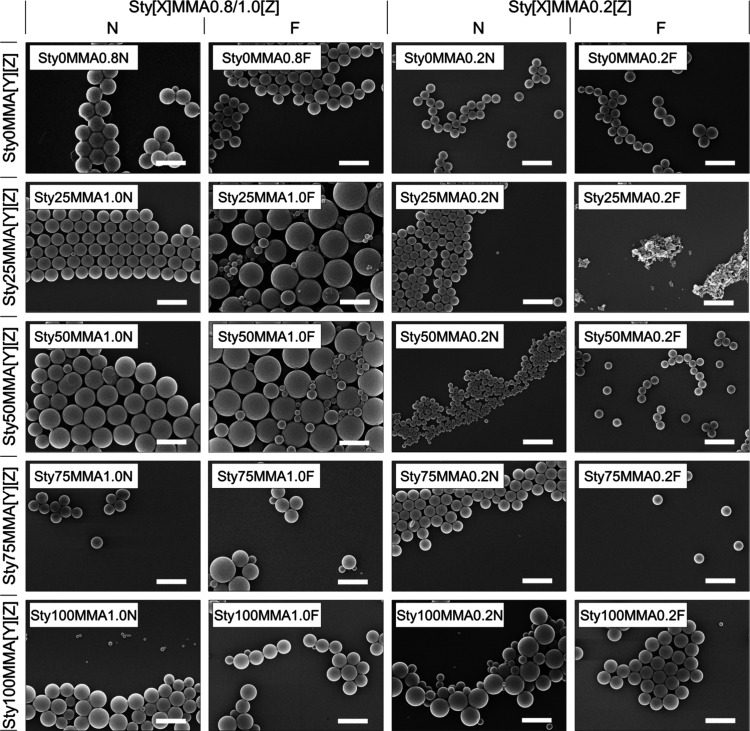
Representative SEM images of synthesized particles for exemplary compositions of the library. Scale bar 1 μm.

Particle size and distribution after dialysis were examined by DLS (Figure 2, data set and 2D versions of the diagrams with the color code are provided in the Supporting Information, Table S2 and Figure S2). To exclude false size data in subsequent analysis, additionally, SEM was performed as an independent method (Figure 3). The comparison of particle diameters from the two different techniques suggested a well dispersed state for most particle compositions, while aggregates were obvious only for a few compositions such as Sty0MMA0.8N (Table 1). An overall trend for the particle size was observed. Generally, the diameter of the particle increased with an increasing ratio of MMA in the feed and increasing monomer concentration in the reaction mixture. DLS indicated particle diameters of around 2000 nm for particles synthesized with low Sty ratios in the feed. This behavior, however, could be attributed to the formation of aggregates during the synthesis as verified by SEM images. Values below 800 nm as measured with DLS were identified as single particles in randomized SEM examinations. DLS measurements also revealed high particle dispersities (increased Polydispersity Index (PdI)) for compositions with high MMA ratios, which is assumed to be a consequence of aggregate formation. Low dispersities were obtained at a high to medium Sty ratio and a low to medium monomer concentration, which is a similar trend as observed for the particle sizes. Interestingly, polyMMA latex beads from industrial production were also previously reported to contain firmly fused aggregates, which may be removed by suitable fractionation techniques.^62^ In the present case, however, the particle appearance in SEM did not indicate any fused particles, suggesting that the samples in fact are nanoparticles and may subsequently be recognized as such in endocytosis/phagocytosis. No general overall trend was observed for the zeta potential of the particles of the library.

**Table 1:** Comparison of Particle Diameters Determined from SEM Images (*d(SEM)*) and DLS (*d(DLS)*) with *r* as the Ratio *d(DLS)*/*d(SEM)*^d^

ID	*d(SEM)* [nm]	*d(DLS)* [nm]	*r*
Sty0MMA0.8N	500	1469	2.9^b^
Sty0MMA0.8F	400	1800	4.5^b^
Sty0MMA0.2N	300	1509	5.0^b^
Sty0MMA0.2F	500	401	0.8
Sty25MMA1.0N	500	555	1.1
Sty25MMA1.0F	800	460	0.6^c^
Sty25MMA0.2N	300	358	1.2
Sty25MMA0.2F	-a	238	-
Sty50MMA1.0N	600	706	1.2
Sty50MMA1.0F	800	490	0.6^c^
Sty50MMA0.2N	200	189	0.9
Sty50MMA0.2F	250	259	1.0
Sty75MMA1.0N	400	398	1.0
Sty75MMA1.0F	500	407	0.8
Sty75MMA0.2N	400	409	1.0
Sty75MMA0.2F	350	237	0.7^c^
Sty100MMA1.0N	600	561	0.9
Sty100MMA1.0F	700	420	0.6^c^
Sty100MMA0.2N	500	505	1.0
Sty100MMA0.2F	500	308	0.6^c^

a Agglomerates, no single particles.

b *r* > 1.2 indicates aggregate formation during DLS measurements.

c *r* < 0.8 indicates overapproximation of the particle size due to broad particle size dispersity.

d SEM: number-weighted distribution; DLS: intensity-weighted distribution.

### 3.2 Multivariant Data Analysis of Physicochemical Particle Characteristics

To understand the interplay and correlations of the physicochemical particle properties, a principal component analysis (PCA) was performed based on the variables according to Table 2. The PdI was observed to be not an independent parameter (Figure S4 in the Supporting Information) and therefore was not included as a variable for PCA. The first two principal components (PC1 and PC2), covering ∼75% of the data (for loadings see Table 2), were considered for further discussion.

**Table 2:** Variables for PCA of the Particulate System and Their Loadings for PC1 and PC2

			loadings	
Abbr.	Variable/description	unit	PC1 (43.1%)	PC2 (31.7%)
*c*	Monomer concentration (feed)	mol·L^–1^	–0.21	0.66
*x*	Molar ratio of Sty in the copolymer (NIR)	mol %	0.54	0.34
	Number-average of molecular weight (GPC)	kg·mol^–1^	–0.62	–0.02
*d*	Particle diameter (DLS)	nm	–0.50	–0.09
*-z*	Transformed zeta potential^a^	mV	–0.15	0.66

a Measured zeta potential values were negative (anionic surfaces). To allow handling of all variables in PCA with a positive sign, the zeta potential data were transformed by multiplication with −1 (−*z*).

**Figure 4:**
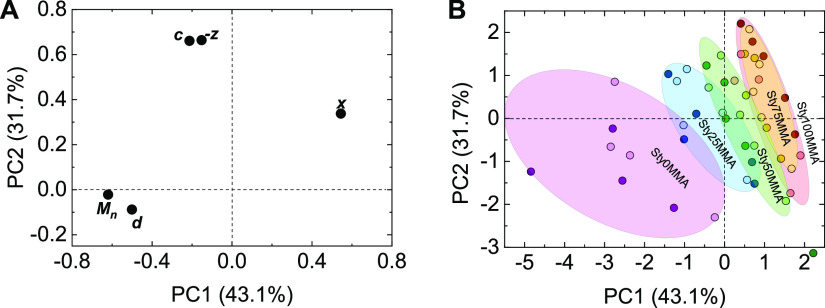
(A) Loading plot of PCA. (B) Score plot of PCA. Clouds indicating data of particles with similar feed composition. Bright colored dots: fluorescence-labeled particles; darker colored spots: nonlabeled particles.

**Figure 5:**
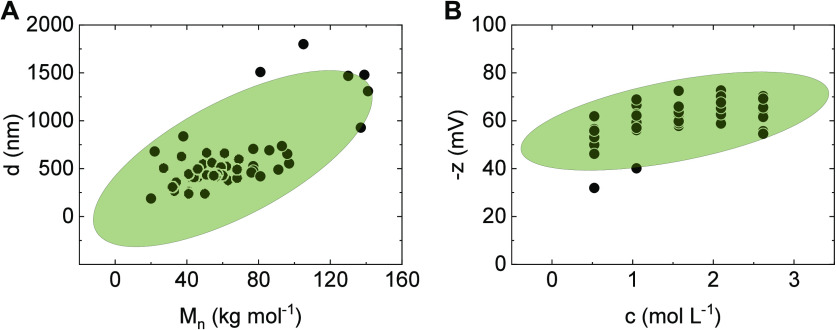
Direct correlations between (A) particle diameter (d) and number-average molecular weight () and (B) negative zeta potential (−*z*) and feed concentration (c) with mean confidence ellipses (95%). Aggregated particle samples were excluded. Both ellipses indicate a positive trend.

**Figure 6:**
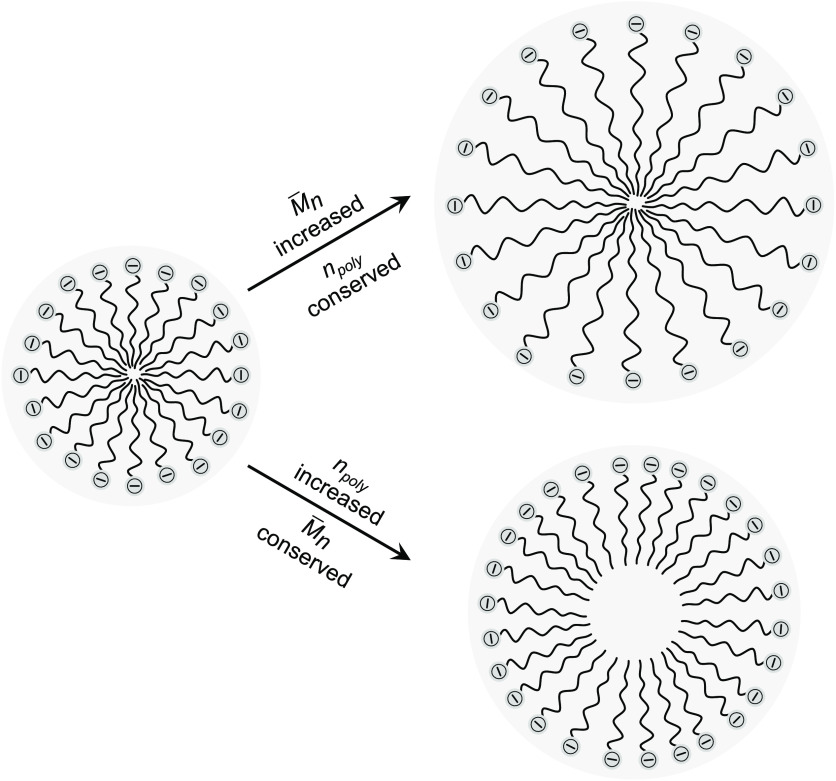
Proposed model for particle size increases during emulsion polymerization by an increase of (i) the number-average molecular weight  of the initiated polymer chains or (ii) the number of polymer molecules per particle *n*_*poly*_ by further chain initiation events. The schematic images show both boundary cases; the central holes are artifacts of simple depiction and do not reflect reality.

The loading plots (Figure 4A) showed two clusters of variables formed by *c* and −*z* as well as *d* and , indicating that these variable pairs are directly correlated (as also shown in Figure 5), while *x* was independent. In the score plots (Figure 4B), the samples formed separated data clouds when categorized according to their composition, demonstrating the high loading of the composition *x* for PC1. We hypothesized a simple constitution principle for particles originating from soap-free emulsion polymerizations: the particle diameter increases by increasing molecular weight of the polymers or by the theoretical addition of more polymer molecules to the ideal micellar constructs from which the particles were formed (Figure 6).

The particle diameter *d* is a characteristic property of polymer particles, which is typically considered to be highly relevant for biointeractions such as cellular uptake. In this context, a multivariate correlation of *d* with −*z* and  as independent variables, picked from the variable clusters, was performed, assuming an ideal micellar structure during synthesis.^63^ As an approximation, according to the proposed model, charges should be exclusively located at the micelle surface and be derived from the initiator APS, with each charged moiety being the starting point of a growing polymer chain.^64^ Besides, a direct relationship between the surface charge density and the surface potential is hypothesized^65^ (for physical background and basic equations see the Supporting Information).

The combination of Supp. eqs 2 to 6 leads to eqs 1 and 2, which indicate a simple relationship among *d*, *z*, and  and include the proportionality factor *k* as derived in Supp. eq 7.
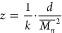
1
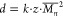
2

**Figure 7:**
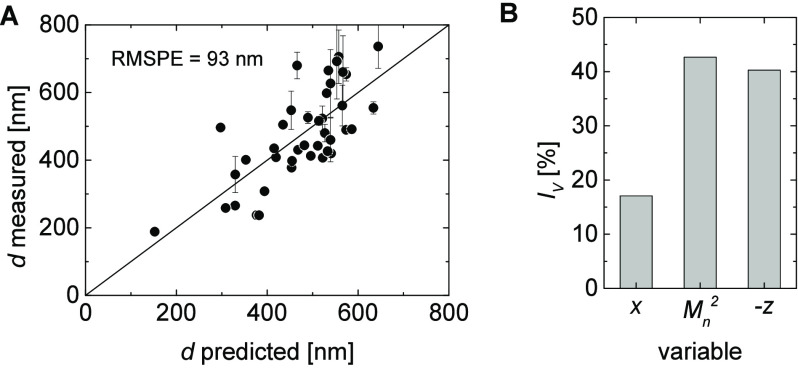
Comparison of measured and predicted particle diameter (*d*) by multiple linear regression (A) (error bars indicate the error from particle size measurements by DLS) and relative impact of variables *I*_*v*_ in this prediction (B). *I*_*v*_ is the normalized value of the product of the variable coefficient and the standard deviation for all measurements for the according variable.

Eq 2 reflects the increase of the particle size by the inclusion of more chains (increase of *n*_*poly*_, reflected by the increase of |*z*|) as well as by the increase of the average chain length (reflected by the increase of ) of the polymers that the particles are composed of, which is reflecting our hypothesis. Despite these relationships being based on several assumptions for simplification, they already led to a good prediction of *d* with a root-mean-square error of prediction (RMSPE) below 240 nm based on the analytically determined *z* and  for 80% of available data (aggregated particle samples were excluded). Furthermore, a multivariate correlation of *d* with *x*, −*z,* and  by multiple linear regression (MLR) led to an even a better prediction of *d* with an RMSPE around 90 nm (Figure 7). The relative impact of variables *I*_*v*_ for this prediction emphasizes the high significance of −*z* and ; however, it also indicates that the comonomer ratio in the copolymers *x* has a considerable impact on the particle size (Figure 7).

### 3.3 Cell Studies

In the next step, the particle library should be investigated in a biological setting to evaluate which parameters in nanoparticle design would be relevant for biointeraction, e.g., if material composition alone or in combination with other parameters would affect cellular uptake. To do so, detrimental effects associated with surface-bound or extractable components should be excluded first. No significant reduction of the viability of the employed HEK-Blue-hTLR4 (direct exposure) was observed for all particle types (viability typically ≥ 80%) except Sty0MMA0.6N and Sty0MMA0.2N. Sty0MMA0.6N and Sty0MMA0.2N showed a visible but not highly relevant reduction of live cells (viability ∼ 60%) (Figure S3). Besides direct cytotoxic effects, the potential presence of endotoxins (lipopolysaccharides, LPS) as proinflammatory and immune cell activating contaminants can be significant, as they are able to stimulate a wide range of different cells. Therefore, endotoxins are undesired, as they may impede the investigation of truly material-associated cellular effects. In order to avoid such contamination, special care was taken to keep the endotoxin burden of the particles during synthesis as low as possible by performing a special rinsing procedure of the robotic volumetric transfer system,^59^ applying WFI only as an aqueous medium and using nonpyrogenic/depyrogenated consumables and glassware. As shown in Figure S3, a low-germ production process of the particle library and practically endotoxin-free particles could be demonstrated. Further details can be found in the Supporting Information (Section 2.2.).

**Figure 8:**
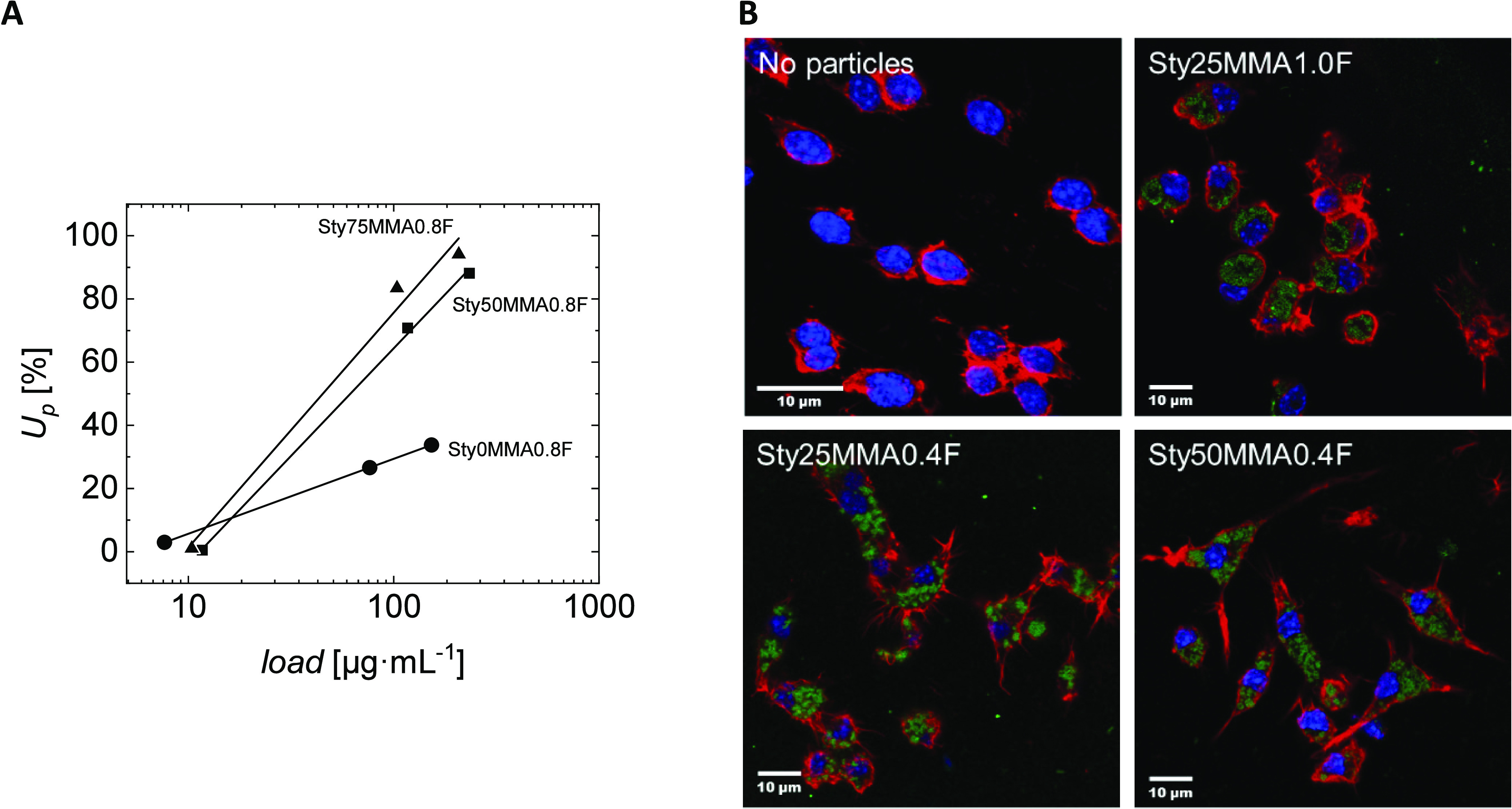
Investigation of the nanoparticle cell interaction. (A) Concentration-dependent nanoparticle uptake by HEK cells as exemplified for samples Sty0MMA0.8F, Sty50MMA0.8F, and Sty75MMA0.8F (for data of all other nanoparticle samples see Table S2 in the Supporting Information). The lines represent the fitting of the data by eq 3 as described in section 3.4. (B) Exemplary confocal laser scanning microscopy analysis of RAW cells treated with particles (5 × 10^5^ cells suspended in 200 μL with 80 μg·mL^–1^ of particles). Exemplary images for selected compositions illustrate the intracellular localization of particles (stained green with fluorescein during synthesis), as observed for all particle types. The negative control (no particles) showed no green structures/particle uptake. Cells were fixated and stained with Alexa Fluor 555 Phalloidin (actin, red fluorescence) and DAPI (nucleus, blue fluorescence).

As the next step of evaluating the biointeraction, nonphagocytic HEK cells and the phagocytic RAW264.7 cells were treated with increasing loads of fluorescence labeled particles to characterize the uptake efficiency by flow cytometry after 24 h. Preliminary experiments showed considerable uptake at a particle load of about 8 μg·mL^–1^ and a beginning saturation at about 160 μg·mL^–1^ (for the gating strategy for differentiation of particle-positive cells in flow cytometry see Figure S4 in the Supporting Information). Thus, these two particle concentrations and an additional particle amount of about 80 μg·mL^–1^ were used subsequently. Three samples (Sty25MMA0.2F, Sty50MMA0.2F, and Sty100MMA0.4F) were excluded, as their low solid content was not sufficient for the planned titration of the cells. The uptake was found to be very high for the phagocytic macrophage-like RAW cells, whereas a low to moderate uptake was observed for nonphagocytic HEK cells. Exemplary plots of the number of nanoparticle positive cells depending on particle concentration are shown in Figure 8A, as quantitatively analyzed in Section 3.4. Additional confocal laser scanning microscopy investigations with fixated and stained cells confirmed that the particles of all compositions were truly located inside the cells, thus supporting the flow cytometry data (exemplary images in Figure 8B and Figure S7 in the Supporting Information). This was ensured by applying a well-established protocol^18,23^ to prepare cells before the flow cytometric measurements in order to exclude all particles, which are just adhering to the cell surface. This included a thorough repeated washing of the cells, as also recognized by the very low number of single particles between the cells. Staining with phalloidin visualizes the cell boundaries (red fluorescence), and all particles of the exemplary micrographs taken within this study were found within these boundaries. Moreover, the particles are always tightly associated with the cell nucleus and stained with DAPI (blue fluorescence).

### 3.4 Multivariant Data Analysis of Biointeraction

To more deeply characterize principles that affect the particle uptake efficiency, the correlation of cellular uptake and nanoparticle concentration in a cell culture was investigated. It was observed that the resulting pattern is not linear but follows a degressive growth kinetics (Figure 8A). Thus, a logarithmic cellular uptake model is proposed. The percentage of particle positive cells *U*_*P*_ was calculated by eq 3 with  as the corrected dimensionless particle load in the cell culture (*p* is the experimental particle load in μg mL^–1^, *p*_0_ is the arbitrary standard particle load of 1 μg mL^–1^) and the uptake parameters *u*_*S*_ (slope) and *u*_*L*_ (level).
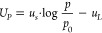
3

Table 3 shows *u*_*s*_, *u*_*L*_, and *U*_*P*_ for the cellular uptake studies with RAW as well as HEK cells as obtained by fitting the plotted data by linear regression according to eq 3. Generally, good correlations with the proposed uptake model (R^2^ > 0.95; Table S2 in the Supporting Information) were observed; samples with correlations R^2^ < 0.80 were excluded from further considerations. While the uptake level was very similar for both types of cells, the uptake slope was much higher for RAW cells, i.e., that the increase of uptake by addition of more particles is higher and saturation is attained considerably faster.

**Table 3:** Uptake Parameter *u*_*S*_ and *u*_*L*_ and Percentage of Particle Positive Cells *U*_*P*_ for a Particle Load of 100 μg·mL^–1^ for the Cellular Uptake Studies with RAW and HEK Cells^d^

	RAW				HEK			
ID	*u*_*S*_[]	*u*_*L*_ [%]	*U*_*P*_ [%]	*Er*^a^ [%]	*u*_*S*_ []	*u*_*L*_ [%]	*U*_*P*_ [%]	*Er*^a^ [%]
Sty0MMA0.8F	51	41	60	4	24	18	29	11
Sty0MMA0.6F	57	45	69	7	43	37	48	10
Sty0MMA0.4F	66	50	82	10	40	32	47	7
Sty0MMA0.2F	67	64	71	10	27	28	26	10
Sty25MMA1.0F	59	46	71	9	35	30	40	11
Sty25MMA0.8F	64	53	75	8	58	54	61	8
Sty25MMA0.6F	70	52	89	10	45	36	54	8
Sty25MMA0.4F	58	46	71	9	43	35	51	10
Sty50MMA1.0F	71	69	72	8	67	74	61	8
Sty50MMA0.8F	74	74	75	8	68	72	64	7
Sty50MMA0.6F	54	58	50	8	24	27	-b	16
Sty50MMA0.4F	65	70	59	7	15	17	-b	12
Sty75MMA1.0F	74	56	92	5	73	60	86	7
Sty75MMA0.8F	72	65	79	5	74	73	76	8
Sty75MMA0.6F	73	72	73	3	74	82	65	8
Sty75MMA0.4F	71	74	69	6	71	85	57	7
Sty75MMA0.2F	73	72	74	12	33	39	-b	7
Sty100MMA1.0F	0.88	0.12	-b	-	13	3.1	-b	9
Sty100MMA0.8F	65	38	92	3	76	64	89	5
Sty100MMA0.6F	69	26	100^c^	8	53	24	81	8
Sty100MMA0.2F	67	49	85	9	67	68	65	6

a Error as expected from the standard deviation of the cellular uptake measurements.

b Correlation with the uptake model with R^2^ < 0.8.

c Calculative value > 100% was set to 100%.

d Base data for this experiment are found in Table S2 of the Supporting Information.

**Figure 9:**
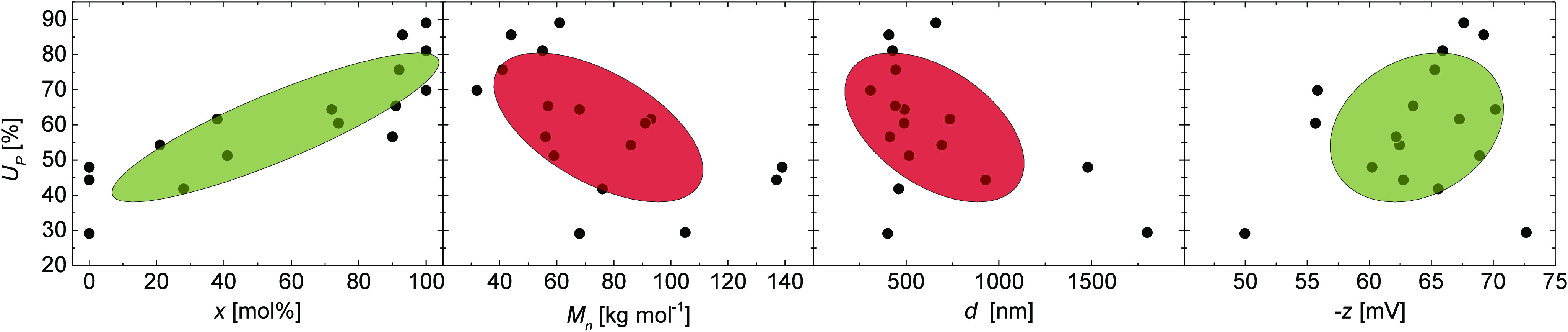
Single parameter correlations for particle uptake in HEK cells with mean confidence ellipses (99.9%). Green ellipses indicate a positive trend; red ellipses indicate a negative trend.

Moreover, the specificity of the uptake by RAW cells regarding the different quality of particles was very low. This has not been a surprising result as RAW cells are reported to have scavenger receptors, which recognize anionic macromolecules and enhance their cellular uptake.^66^ However, significant differences were found for the HEK cells. In general, the ratio of particle positive cells was higher for particles with a higher content in styrene (*x*) as seen in Figure 9. Admitting that hydrophobicity of particulate systems is more complex and cannot be reduced to compositional factors only,^67^ this effect might still be ascribed to the increase of hydrophobicity of the particles (as also demonstrated by water contact angle measurements with thin films obtained by melt-compression of nanoparticles in Figure S2), when the content of styrene is increased, potentially facilitating cell membrane particle interaction. The other single parameter correlations (*U*_*p*_ vs , *d*, and −*z*) are also displayed in Figure 9. The lower aspect ratio of the confidence ellipses indicates a lower significance of these parameters.

Negative trends were found for  as well as *d*. Similar trends were expected, as it was already known that both parameters are in a strong correlation (Figure 4A). This negative trend, however, also indicated that particles with a low diameter are taken up more easily, which is a plausible result for particles in this length scale.^68^ A positive trend with a rather low significance was observed for −*z*. According to the model for the particle size increase (Figure 6), this may lead to the interpretation that particles with a high charge density on their surfaces are taken up preferentially. This was rather surprising as the literature reports that negative charges (as in this case) have a repulsive effect due to the slight negative charge of cell membranes.^69^

**Figure 10:**
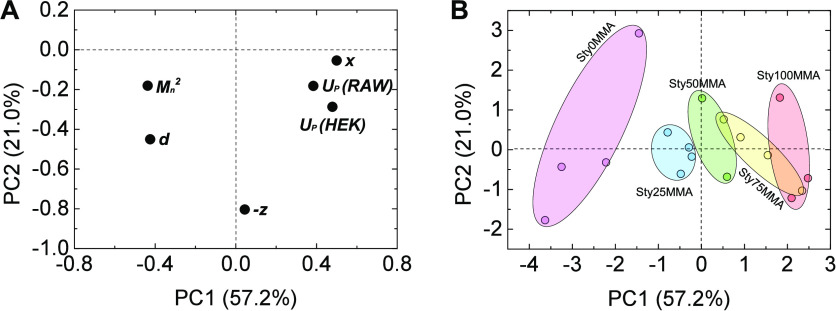
Loading (A) and score plots (B) of principal component analysis of the cellular uptake behavior.

To identify more specific correlations, a PCA was also applied to this data set. The first two principal components (PC1 and PC2) used as data coverage were sufficient (>78%). Table 4 and Figure 10 display loadings and scores of PC1 and PC2.

**Table 4:** Variables for Principal Component Analysis of the Cellular Uptake Behavior and Loadings for PC1 and PC2

			loadings	
Abbr.	Variable/description	unit	PC1 (57.2%)	PC2 (21.0%)
*x*	Molar ratio of Sty in the copolymer (NIR)	mol %	0.50	–0.05
	Squared number-average of molecular weight (GPC)	kg^2^·mol^–2^	–0.44	–0.18
*d*	Particle diameter (DLS)	nm	–0.42	–0.45
–*z*	Transformed zeta potential^a^	mV	0.04	–0.80
*U*_*P*_*(RAW)*	Percentage of particle-positive RAW cells	%	0.38	–0.18
*U*_*P*_*(HEK)*	Percentage of particle-positive HEK cells	%	0.48	–0.29

a Measured zeta potential values were negative (anionic surfaces). In order to allow handling of all variables in PCA with a positive sign, the zeta potential data were transformed by multiplication with −1 (−z).

**Figure 11:**
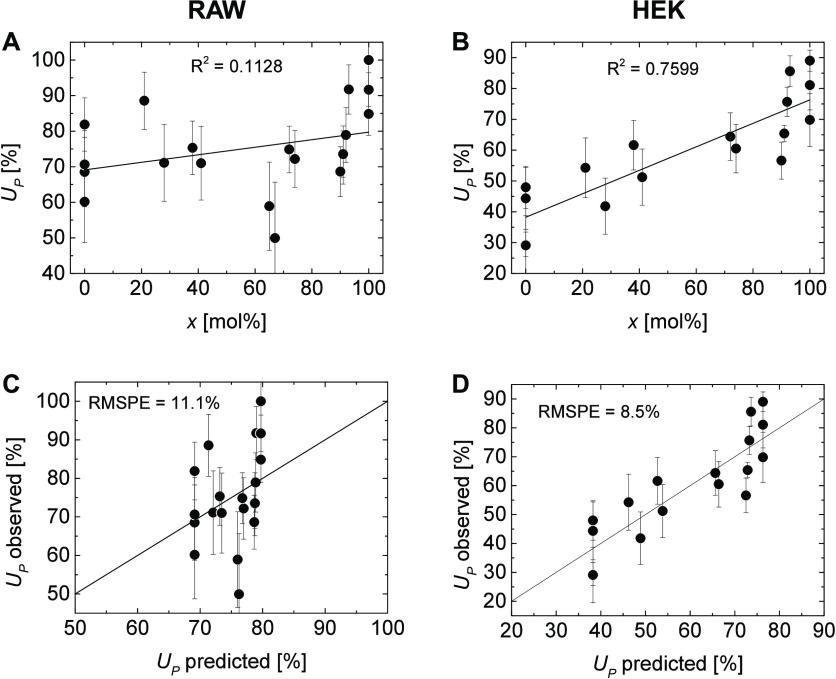
Linear fit of the cellular uptake *U*_*P*_ vs composition *x* of the particles for RAW (A) and HEK cells (B) and the according prediction diagrams for RAW (C) and HEK cells (D). Error bars indicate the expected error from the standard deviation of the cellular uptake measurements.

The observed clustering of *x* and *U*_*P*_ for both cell types in the loading plots (Figure 10A) can be taken as evidence for the importance of the copolymer composition for the cellular uptake of the particles as already deducible from single parameter correlations in Figure 9. When categorized by composition, nearly separated data clouds were obtained in the score plots (Figure 10B), which is another manifestation of the high importance of *x* for the whole system. However, testing for a direct linear correlation of *x* and *U*_*P*_ revealed a very low fitting quality (R^2^ = 0.1128; Figure 11A) with an unacceptably high RMSPE of 11.1% for the correlation of observed and predicted RAW cell uptake (Figure 11C). The average uptake in this set of experiments was 75.4 ± 12.1% (mean ± standard deviation); hence, the RMSPE is more than 90% of the standard deviation, rendering the prediction hardly better than the mean value of the measurements. Considering the generally high uptake activity of phagocytic macrophage-type cells, such a pattern may be justifiable. In the case of the nonphagocytic HEK cells, for which uptake proceeds through endocytosis, a better fitting quality (R^2^ = 0.7599; Figure 11B) and a lower RMSPE of 8.5% (48% of the standard deviation of *U*_*P*_) were observed for a direct linear correlation (Figure 11D).

**Figure 12:**
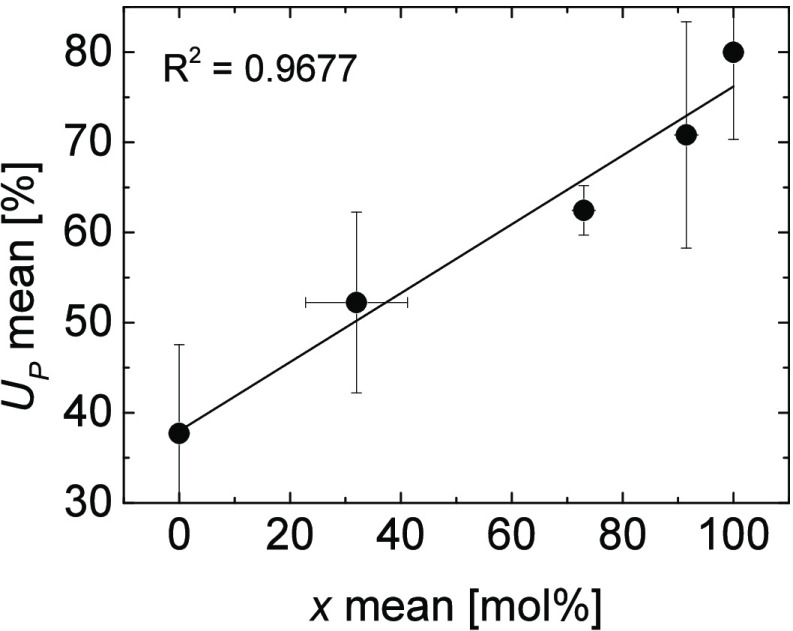
Linear fit of mean values for the cellular uptake *U*_*P*_ and composition *x* of the particles for HEK cells. Error bars indicate the standard deviation of the values used for averaging.

Using the mean of all experimental *x* values for the same nominal Sty feed (e.g., 25 mol %), a very good linear fit (R^2^ = 0.9677) with the likewise averaged corresponding *U*_*P*_ was obtained (Figure 12). Although the large error bars in Figure 12 should be considered when this correlation is applied to predict the behavior of individual samples, the good fit was an unexpected and surprising finding. It is assumed that the complementary influence of other parameters (e.g., , *d*, and −*z*) could advantageously be averaged out by this procedure.

**Figure 13:**
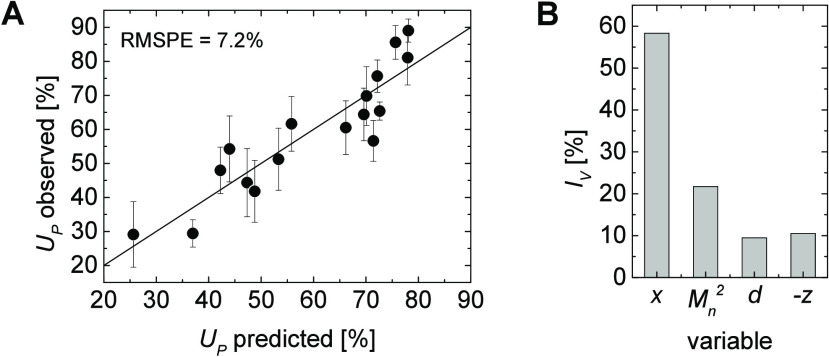
Observed versus predicted cellular uptake *U*_*P*_ of HEK cells by multiple linear regression (A) (error bars indicate the expected error from the standard deviation of the cellular uptake measurements) and relative variable impact *I*_*v*_ for the prediction (B).

These observations encouraged us to conduct a multivariate correlation of the cellular uptake behavior of HEK cells by MLR including the identified complementary parameters , *d*, and −*z* (compare Figure 10A). In this analysis, the RMSPE decreased by 1.3% to 7.2% (Figure 13A). The impact of the variables basically followed the same trend as that found for the single parameter consideration in Figure 9. The composition, *x*, had, as expected, the highest impact, moreover, using  instead of  for these calculations again delivered better results, which endorsed the proposed relationship among *d*, *z*, and  (Figure 13B).

## 4 Conclusion

Particulate systems with a specific behavior toward cells are promising systems for targeted administration of therapeutics, including anticancer drugs and next-generation vaccines.^6^ By employing a model copolymer particle library and a thorough characterization, we could show differences in their cellular uptake behavior, including differences between macrophage-derived phagocytic cells and nonphagocytic HEK cells, which might better mimic the behavior expectable for body cells that are not specialized on engulfing foreign substances. Unspecific particle uptake was observed for the phagocytic cells, while nonphagocytic cells showed an uptake behavior dependent on the physicochemical properties of the particles. By multivariate analysis, the contribution of particle features affecting uptake, specifically single parameters like composition, to the overall effect could be elucidated, and a model for the prediction of the cellular uptake was proposed. Other commonly stressed parameters, such as size or charge, were contributing only to a lower or marginal extent, respectively, according to principal component analysis. It should be noted that such predictions, as always for PCA and other statistical analyses, should not be understood as generally applicable causalities. Especially in biological systems, many underlying variables, which also influence cellular behavior, remain unconsidered or are even unknown. Still, the presented approach here shows how structure–property relationships in a multiparameter design space (particle composition, size, charge, etc.) can be elucidated for cellular uptake as a complex physiological process. The approach may facilitate the efficient design of nanocarriers for intracellular delivery of substances and cell modulation.

## Supporting Information Available

Additional experimental details on Materials and Methods, High-Throughput Synthesis, Characterization, Dialysis of the Polymer Library, Confocal Laser Scanning Microscopy, Hot-press Molding, Water Contact Measurements. Numeric results and 2D graphs of polymer and particle characterization, and cellular uptake study (both cell types). Additional details on structure-properties relationships in particle library. Biocompatibility (cytotoxicity and endotoxin burden) of particle library. Gating strategy for flow cytometry and uptake studies. Additional confocal laser scanning micrographs (particle/cell interaction) (PDF)
Machine readable (comma-separated value/csv format) result data of particle characterization (x, m.w., d, PdI, zeta) and uptake study (RAW and HEK cells) (TXT)
