# Supplementary material for: Multivariate Analysis of Cellular Uptake Characteristics for a (Co)polymer Particle Library
Source: ACS Biomater Sci Eng. 2024 Feb 20;10(3):1481–93. doi: 10.1021/acsbiomaterials.3c01803 (PMC10934412; doi:10.1021/acsbiomaterials.3c01803)
Supplement: Supplementary file 1 — ab3c01803_si_001.pdf [file ab3c01803_si_001.pdf]

Supporting Information

Multivariate analysis of cellular uptake characteristics for a (co)polymer particle library

Stefan Baudis,[1,2] Toralf Roch,[1,3] Maria Balk,[1] Christian Wischke,[1,4] Andreas Lendlein,[1,5] Marc Behl[1*]*

[1] *Institute of Active Polymers, Helmholtz-Zentrum Hereon, Kantstraße 55, 14513 Teltow, Germany*

[2] *Current address: Institute of Applied Synthetic Chemistry, Technische Universität Wien, Getreidemarkt 9/163MC, 1060 Vienna, Austria*

[3] *Current address: CheckImmune GmbH, Campus Virchow Klinikum, Augustenburger Platz 1, 13353 Berlin, Germany*

[4] *Current address: Institute of Pharmacy, Martin-Luther University Halle-Wittenberg, Kurt-Mothes-Straße 3, 06120 Halle (Saale), Germany*

[5] *Institute of Biochemistry and Biology, University of Potsdam, Karl-Liebknecht-Str. 24-25, 14476 Potsdam-Golm, Germany*

** Corresponding authors:*
S. Baudis (stefan.baudis@tuwien.ac.at),
C. Wischke (christian.wischke@hereon.de, christian.wischke@pharmazie.uni-halle.de),
M. Behl (marc.behl@hereon.de)

Content

1. Materials and Methods 2
   1.1. Materials 2
   1.2. High Throughput Synthesis of the Polymer Library 2
   1.3. High Throughput Characterization of the Particle Library 4
   1.4. Dialysis of the Particle Library 5
   1.5. Confocal Laser Scanning Microscopy 5
   1.6. Hot-press molding of polymer disc specimens and water contact angle measurements 5
2. Results 6
   2.1. Structure-properties relationships in particle library 9
   2.2. Biocompatibility of Synthesized Particle Library 10
   2.3. Gating Strategy for the Flow Cytometry and Uptake Studies 11
3. References 14

1. Materials and Methods

1.1. Materials

Monomers were purchased from Sigma-Aldrich (Steinheim, Germany). Methyl methacrylate (MMA, ≥ 99%, Fluka) was freshly distilled prior use. Styrene (Sty, ≥ 99%) was extracted three times with 10% aqueous sodium hydroxide solution, dried over anhydrous magnesium sulfate, and distilled *in vacuo* (and stored at -20 °C unless not polymerized immediately). Fluorescein-O-acrylate (FA, 95%) was used as solution in ethanol (quality for molecular biology) in a concentration of 4 mg/mL. Ammonium persulfate (APS, > 98%, Merck, Darmstadt, Germany) was recrystallized from an ethanol/water mixture and used as aqueous solution with a concentration of 34 mg/mL. Ammonium bicarbonate (ABC, > 99%, Bernd Kraft, Duisburg, Germany) was used as aqueous solution with a concentration of 71 mg/mL. Water used to prepare the solutions and as reaction- and washing medium was of "water for injection" quality (WFI) (Thermo Scientific HyClone HyPure WFI; provider: Fisher Scientific, Schwerte, Germany).

1.2. High Throughput Synthesis of the Polymer Library

Particles were synthesized by soap-free emulsion polymerization (Scheme S 1). All monomers (incl. the ethanolic solution of FA) were purged with nitrogen for at least 5 min and all aqueous solutions were evacuated and vented with nitrogen three times to remove dissolved oxygen. The reactors were pretreated at 140 °C under vacuum for 45 min followed by three cycles (each 10 min) of evacuation and venting with nitrogen at the same temperature. Water (volume adapted to have a total volume of 3.3 mL of the continuous phase) was loaded volumetrically to each reaction vessel and degassed at 20 °C (3 cycles á 5 min while vortexing). ABC buffer solution (0.1 mL) and APS initiator solution (0.1 mL) were added. Reactors were heated to 80 °C for 30 min and cooled to 15 °C. Then, Sty, MMA (in sum 8.64, 6.91, 5.18, 3.46, and 1.72 mmol) and FA solution (calculated for 0.2 wt% with respect to the total amount of monomer) were added volumetrically (densities used for MMA: 0.940 $g \cdot mL^{-1}$, Sty: 0.910 $g \cdot mL^{-1}$). Another portion of APS initiator solution (calculated for 787 $\mu g \cdot mmol^{-1}$ monomer) was added and the reactors were vortexed at 1000 rpm for Sty-MMA copolymerization at 80 °C for 3 h. Subsequently, a third portion of APS initiator solution (0.2 mL) was added to complete reaction (1000 rpm, 80 °C, 3 h). A schematic overview of the procedure is shown in Scheme S1.

Generally, the obtained particle suspensions were only handled with sterile/non-pyrogenic consumables or with glassware that was heated to 185 °C for least one hour.

An overview of all synthesized particle is presented in Table S 1.

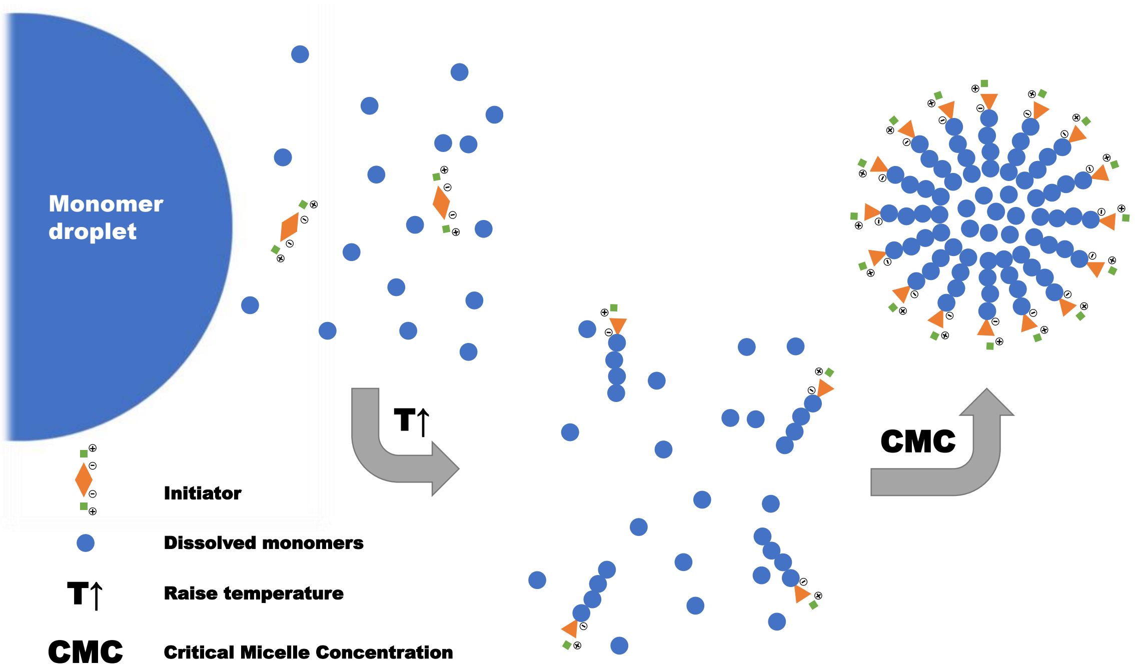

Scheme S 1. Schematic of soap-free emulsion polymerization. Radical initiation occurs in the aqueous phase and amphiphilic polymer chains (ionic/polar head from initiator with non-polar polymer chain) are formed, which assemble to micelles above the critical concentration CMC. Polymerization continues within the micelles by inclusion of more monomers from the aqueous phase.

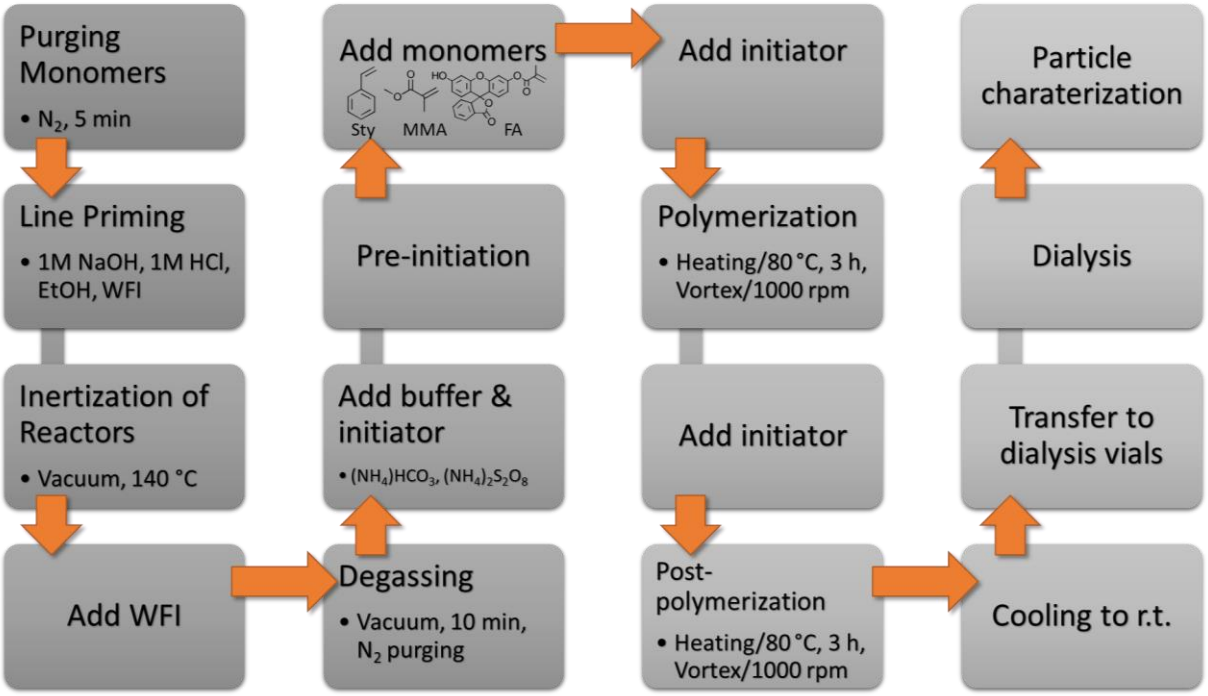

Scheme S 2. Schematic procedure of polymer particle synthesis from styrene (Sty), methyl methacrylate (MMA) and fluorescein-O-acrylate (FA) by soap-free emulsion polymerization using water for injection (WFI).

Table S 1. Denotation of the particles of the synthesized library based on feed composition

	monomer conc. [mmol·mL^{-1}]	conc. factor [ ]	Sty in the feed [mol%] 0	25	50	75	100
non-labelled	2.62	1.0	-[a)]	Sty25MMA1.0N	Sty50MMA1.0N	Sty75MMA1.0N	Sty100MMA1.0N
	2.10	0.8	Sty0MMA0.8N	Sty25MMA0.8N	Sty50MMA0.8N	Sty75MMA0.8N	Sty100MMA0.8N
	1.57	0.6	Sty0MMA0.6N	Sty25MMA0.6N	Sty50MMA0.6N	Sty75MMA0.6N	Sty100MMA0.6N
	1.05	0.4	Sty0MMA0.4N	Sty25MMA0.4N	Sty50MMA0.4N	Sty75MMA0.4N	Sty100MMA0.4N
	0.52	0.2	Sty0MMA0.2N	Sty25MMA0.2N	Sty50MMA0.2N	Sty75MMA0.2N	Sty100MMA0.2N
fluores-cence-labelled	2.62	1.0	-[a)]	Sty25MMA1.0F	Sty50MMA1.0F	Sty75MMA1.0F	Sty100MMA1.0F
	2.10	0.8	Sty0MMA0.8F	Sty25MMA0.8F	Sty50MMA0.8F	Sty75MMA0.8F	Sty100MMA0.8F
	1.57	0.6	Sty0MMA0.6F	Sty25MMA0.6F	Sty50MMA0.6F	Sty75MMA0.6F	Sty100MMA0.6F
	1.05	0.4	Sty0MMA0.4F	Sty25MMA0.4F	Sty50MMA0.4F	Sty75MMA0.4F	Sty100MMA0.4F
	0.52	0.2	Sty0MMA0.2F	Sty25MMA0.2F	Sty50MMA0.2F	Sty75MMA0.2F	Sty100MMA0.2F

[a)] experiments were omitted to have 48 reactions (in 3 x 16 reactor arrays)

1.3. High Throughput Characterization of the Particle Library

DRIFT measurements were done with a Bruker Vertex 70 spectrometer (Bruker Optik, Ettlingen, Germany) with the high throughput extension HTS-XT for microwell plate support under dry and carbon dioxide-free atmosphere (FT-IR Purge gas generator, Parker/Balston, Haverhill, MA, USA). The particle suspensions were transferred into the microwell plate and dried overnight in the vacuum oven at 40 °C before DRIFT measurements were performed. Bruker Optik OPUS 7.0 software with Quant 2 spectra analysis tool was used to calculate the composition of the dried particles on the basis of their NIR spectra as described previously.[1]

The HT GPC was combined with a PSS Universal Data Center (PSS, Mainz, Germany). Two serially operated HT-GPC columns type PSS SDV analytical linear M 5 µm (PSS, Mainz, Germany), THF as eluent (35 °C, flow rate 1.0 mL·min^{-1}) with 0.05 wt% 3,5-di-tert-butyl-4-hydroxytoluene (Sigma-Aldrich, Steinheim, Germany) as internal standard, and polystyrene standards (PSS, Mainz, Germany) were used for standard calibration to determine the number average of the molecular weight ($\overline{M}_n$) and the polydispersity (P_d). PSS WinGPC Unity software enabled overlapped sample injection to increase throughput. GPC sample preparation was carried out semi-automatically employing the robotic platform Accelerator SLTII/106 (Chemspeed Technologies, Augst, Switzerland) as described before.[2]

The HT determination of solid content in each particle suspension volume was performed with dialyzed samples in a semi-automatic procedure employing the robotic platform Accelerator SLTII/106 (Chemspeed Technologies, Augst, Switzerland) by drying to constant mass. Vials were tared within the robotic platform and 50 µL of each homogenized latex were transferred into them manually. Subsequently, the samples were dried for 48 h under a mild air stream at ambient temperature and the vials were weighed within the platform to calculate the dry mass of the samples. Drying and weighing were repeated until constant masses were obtained.

1.4. Dialysis of the Particle Library

500 µL of (homogenized) latices were transferred into the filter units that were placed in a rack of 8 mL glass vials (Chemspeed Technologies, Augst, Switzerland) filled with about 7.5 mL WFI to ensure full wetting of the membrane but leave an air bubble to enhance convection during shaking. The rack was shaken for time periods up to 14 days employing an IKA HS 501 Digital Horizontal Shaker (IKA, Staufen, Germany) at 160 to 190 rpm. The progress of dialysis was monitored by fluorescence spectroscopy using a Varian Cary Eclipse Fluorescence Spectrophotometer (Agilent Technologies, Böbligen, Germany) by standard calibration (0.0, 0.4, 1.0, 2.0, 4.0, 10.0, and 20.0 ppm of fluorescein o-acrylate in water) at an excitation wavelength of 486 nm and an emission wavelength of 513 nm. A decrease of the concentration of extractable fluorescein-O-acrylate below 1 ppm has been defined as the end point of dialysis (mean concentration of all samples after dialysis was < 0.2 ppm).

1.5. Confocal Laser Scanning Microscopy

Microscopy of RAW cells was performed as described previously [3, 4]. Briefly, to visualize the uptake of particles, RAW cells were fixated with 4% paraformaldehyde, stained with 400 $\mu g \cdot ml^{-1}$ Alexa- fluor 555 Phalloidin (actin) and 10 $\mu g \cdot ml^{-1}$ DAPI (nucleus), and analyzed by confocal laser scanning microscopy (CLSM; LSM 510 META on Axiovert 200M, Zeiss, Jena, Germany).

1.6. Hot-press molding of polymer disc specimens and water contact angle measurements

For preparation of polymer disc specimens with a diameter of 4.7 mm and a thickness of about 1 mm, the copolymer was first homogenized by milling, subsequently about 20 mg for each specimen of the copolymers were inserted into a custom-made tool and hot-pressed using the following pressure/temperature program: (1) 150 °C without load for 60 s, (2) 2000 kg load at 150 °C for 60 s, (3) cooling down phase at 2000 kg load for 120 s.

Water contact angles (WCA) were measured goniometrically (image acquisition with a digital microscope, evaluation was performed by means of the graphical software imageJ) using the sessile drop technique with distilled water as test liquid. About 20 µL of water were dispensed onto polymer disc surfaces. The diameter of the contact areas between surface and drop was always bigger than 2 mm. At least 4 measurements were averaged to calculate each WCA value and standard deviation.

2. Results

Table S 2. Cellular uptake study: particle load and according particle positive cells (U_p) with calculated uptake parameter u_s and u_p (and correlation coefficient R^2).

		HEK				RAW			
**SampleID**	***load* [µg/mL]**	**U_p [%]**	**u_s**	**u_L**	**R^2**	**U_p [%]**	**u_s**	**u_L**	**R^2**
Sty0MMA0.8F	7,6	3,0				2,8			
	76,4	26,6				44,3			
	152,9	33,8	23,7	18,0	1,000	72,1	50,3	43,3	0,966
Sty0MMA0.6F	11,4	8,2				10,1			
	113,9	47,5				74,5			
	227,9	64,9	42,5	37,4	0,994	86,7	60,3	52,9	0,995
Sty0MMA0.4F	7,5	2,9				3,5			
	75,0	40,5				79,1			
	149,9	55,8	39,9	32,4	0,996	89,3	68,4	55,0	0,987
Sty0MMA0.2F	9,1	0,2				0,8			
	90,8	15,2				66,2			
	181,7	39,9	26,7	27,6	0,818	89,4	67,3	64,1	0,999
Sty25MMA1.0F	6,5	0,1				0,4			
	64,7	24,3				58,4			
	129,4	50,7	35,1	30,4	0,898	78,9	59,8	48,4	0,999
Sty25MMA0.8F	9,8	2,8				4,7			
	97,9	62,1				81,7			
	195,9	77,5	57,9	54,4	0,999	88,8	67,7	60,6	0,979
Sty25MMA0.6F	5,9	0,2				1,8			
	59,1	37,9				73,7			
	118,3	62,2	45,1	36,0	0,969	92,8	70,4	52,2	0,999
Sty25MMA0.4F	5,9	0,2				1,2			
	58,8	31,8				42,9			
	117,7	61,5	43,2	35,2	0,922	81,7	56,8	45,3	0,924
Sty25MMA0.2F	6,7	0,1				0,2			
	67,1	0,2				13,0			
	134,3	0,8	0,4	0,3	0,507	73,6	45,4	43,5	0,623
Sty50MMA1.0F	13,8	1,5				2,5			
	138,0	74,9				92,2			
	275,9	86,3	67,2	74,0	0,990	95,5	76,0	81,6	0,964
Sty50MMA0.8F	11,7	0,5				0,9			
	116,9	70,8				87,2			
	233,8	88,1	68,1	71,8	0,999	96,6	76,8	79,3	0,982
Sty50MMA0.6F	10,5	0,2				0,3			
	105,4	9,3				27,9			
	210,8	37,4	23,7	26,8	0,693	77,3	51,2	56,5	0,800

		HEK				RAW			
**SampleID**	***load* [µg/mL]**	**U_p [%]**	**u_s**	**u_L**	**R^2**	**U_p [%]**	**u_s**	**u_L**	**R^2**
Sty50MMA0.4F	12,9	0,1				0,3			
	129,4	7,7				72,7			
	258,7	22,2	14,6	17,5	0,786	85,4	67,1	73,3	0,993
Sty50MMA0.2F	6,9	0,1				0,2			
	69,4	0,4				20,5			
	138,8	1,6	0,9	0,8	0,614	69,9	45,2	42,5	0,738
Sty75MMA1.0F	7,2	1,2				2,1			
	71,8	79,6				87,9			
	143,7	93,6	72,9	60,2	0,993	98,1	76,8	61,9	0,984
Sty75MMA0.8F	10,4	1,0				3,2			
	103,7	83,4				90,6			
	207,5	94,2	74,3	72,9	0,986	97,0	75,9	71,8	0,974
Sty75MMA0.6F	13,5	0,3				3,8			
	134,5	79,2				94,9			
	269,1	93,7	73,5	81,7	0,994	98,5	77,4	80,9	0,965
Sty75MMA0.4F	16,2	0,3				1,6			
	162,4	73,6				91,3			
	324,9	91,3	70,8	85,0	0,999	99,3	78,7	91,7	0,978
Sty75MMA0.2F	11,5	0,1				1,8			
	115,4	13,2				81,5			
	230,7	51,9	33,1	38,8	0,702	97,4	75,1	77,1	0,996
Sty100MMA1.0F	1,4	0,1				0,2			
	14,4	0,5				3,0			
	28,8	1,5	0,9	0,1	0,699	13,4	8,3	2,1	0,658
Sty100MMA0.8F	8,8	5,5				16,7			
	87,5	95,7				98,0			
	175,0	98,5	76,2	63,6	0,962	99,7	68,2	45,1	0,958
Sty100MMA0.6F	2,5	0,1				1,9			
	25,2	35,5				70,2			
	50,3	76,2	52,7	24,2	0,888	91,8	68,9	25,8	1,000
Sty100MMA0.4F	3,2	0,1				1,5			
	32,4	33,6				74,0			
	64,7	82,7	56,0	32,6	0,842	93,1	71,0	34,4	0,999
Sty100MMA0.2F	9,7	0,3				15,4			
	97,0	53,9				91,6			
	194,1	92,4	66,5	67,8	0,958	98,3	66,9	48,8	0,978

Table S 3. Particle characterization and cellular uptake data.

Variable	conc.	Sty in	Sty out	$\overline{M_n}$	$\overline{M_w}$	Pd (GPC)	d (DLS)	PdI (DLS)	z	U_p (RAW)	U_p (HEK)
**Unit**	*mol L⁻¹*	*mol%*	*mol%*	*kg mol⁻¹*	*kg mol⁻¹*		*nm*		*mV*	*%*	*%*
**SampleID**											
Sty0MMA0.8N	2.09	0	0	130	367	2.82	1469	0.949	-60	N/A	N/A
Sty0MMA0.6N	1.57	0	0	111	332	2.99	5726	1.000	-59	N/A	N/A
Sty0MMA0.4N	1.05	0	0	141	357	2.53	1309	0.826	-56	N/A	N/A
Sty0MMA0.2N	0.52	0	0	81	224	2.77	1509	0.884	-54	N/A	N/A
Sty25MMA1.0N	2.62	25	28	97	238	2.45	555	0.203	-65	N/A	N/A
Sty25MMA0.8N	2.09	25	29	77	193	2.5	526	0.237	-59	N/A	N/A
Sty25MMA0.6N	1.57	25	28	96	237	2.47	654	0.402	-58	N/A	N/A
Sty25MMA0.4N	1.05	25	28	33	84	2.54	266	0.137	-56	N/A	N/A
Sty25MMA0.2N	0.52	25	27	34	128	3.76	358	0.065	-56	N/A	N/A
Sty50MMA1.0N	2.62	50	69	77	187	2.43	706	0.095	-62	N/A	N/A
Sty50MMA0.8N	2.09	50	74	69	177	2.56	598	0.029	-63	N/A	N/A
Sty50MMA0.6N	1.57	50	67	77	171	2.22	480	0.083	-59	N/A	N/A
Sty50MMA0.4N	1.05	50	64	63	147	2.33	378	0.039	-57	N/A	N/A
Sty50MMA0.2N	0.52	50	69	20	36	1.81	189	0.077	-32	N/A	N/A
Sty75MMA1.0N	2.62	75	90	43	85	1.97	398	0.016	-62	N/A	N/A
Sty75MMA0.8N	2.09	75	94	51	118	2.31	665	0.035	-68	N/A	N/A
Sty75MMA0.6N	1.57	75	88	62	151	2.44	523	0.066	-63	N/A	N/A
Sty75MMA0.4N	1.05	75	91	49	123	2.51	548	0.057	-59	N/A	N/A
Sty75MMA0.2N	0.52	75	91	46	114	2.47	409	0.023	-56	N/A	N/A
Sty100MMA1.0N	2.62	100	100	54	160	2.96	561	0.208	-70	N/A	N/A
Sty100MMA0.8N	2.09	100	100	38	119	3.14	837	0.258	-71	N/A	N/A
Sty100MMA0.6N	1.57	100	100	37	98	2.66	627	0.465	-72	N/A	N/A
Sty100MMA0.4N	1.05	100	100	22	39	1.76	680	0.222	-67	N/A	N/A
Sty100MMA0.2N	0.52	100	100	27	124	4.6	505	0.237	-62	N/A	N/A
Sty0MMA0.8F	2.09	0	0	105	282	2.69	1800	0.986	-73	60	29
Sty0MMA0.6F	1.57	0	0	139	328	2.36	1479	0.932	-60	69	48
Sty0MMA0.4F	1.05	0	0	137	299	2.18	928	0.700	-63	82	47
Sty0MMA0.2F	0.52	0	0	68	263	3.87	401	0.101	-50	71	26
Sty25MMA1.0F	2.62	25	28	76	226	2.97	460	0.357	-66	71	40
Sty25MMA0.8F	2.09	25	38	93	226	2.43	736	0.676	-67	75	61
Sty25MMA0.6F	1.57	25	21	86	206	2.39	692	0.203	-62	89	54
Sty25MMA0.4F	1.05	25	41	59	162	2.74	516	0.300	-69	71	51
Sty25MMA0.2F	0.52	25	32	50	149	2.97	238	0.092	-57	N/A	N/A
Sty50MMA1.0F	2.62	50	74	91	168	1.85	490	0.300	-56	72	61
Sty50MMA0.8F	2.09	50	72	68	143	2.11	491	0.066	-70	75	64
Sty50MMA0.6F	1.57	50	67	60	140	2.33	431	0.037	-60	50	N/A
Sty50MMA0.4F	1.05	50	65	51	126	2.47	435	0.022	-57	59	N/A
Sty50MMA0.2F	0.52	50	73	41	108	2.64	259	0.041	-46	N/A	N/A
Sty75MMA1.0F	2.62	75	93	44	99	2.25	407	0.214	-69	92	86
Sty75MMA0.8F	2.09	75	92	41	94	2.3	444	0.151	-65	79	76
Sty75MMA0.6F	1.57	75	91	57	125	2.19	443	0.019	-64	73	65
Sty75MMA0.4F	1.05	75	90	56	129	2.31	413	0.013	-62	69	57
Sty75MMA0.2F	0.52	75	91	41	116	2.83	237	0.100	-53	74	N/A
Sty100MMA1.0F	2.62	100	100	81	376	4.64	420	0.184	-55	N/A	N/A
Sty100MMA0.8F	2.09	100	100	61	181	2.96	660	0.169	-68	92	89
Sty100MMA0.6F	1.57	100	100	55	196	3.57	426	0.319	-66	100	81
Sty100MMA0.4F	1.05	100	100	46	104	2.25	497	0.058	-40	N/A	N/A
Sty100MMA0.2F	0.52	100	100	32	141	4.42	308	0.155	-56	85	65

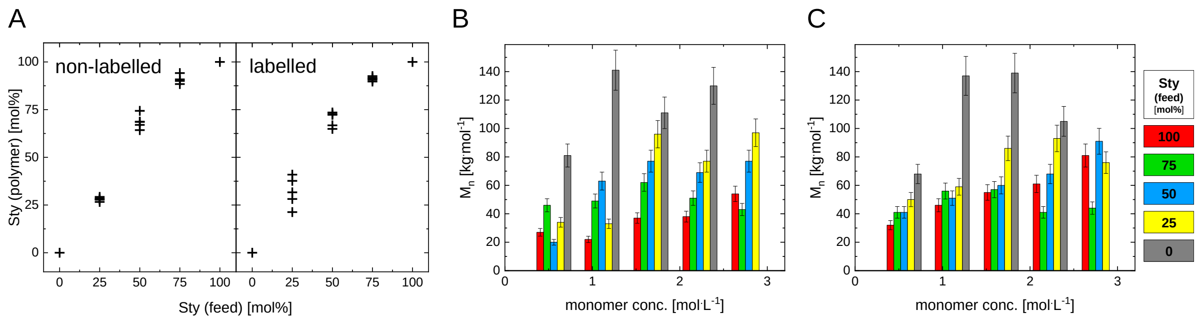

Figure S 1. Results of the high throughput characterization of the particle library: (A) Particle composition of the whole library based on NIR data compared to feed ratios (data for a given feed include all samples synthesized at different monomer concentrations for the same monomer ratios). Error bars for the composition were omitted for clarity reasons (characteristic error ± 2 mol%). (B) Molecular weight of non-labelled polymer particles (GPC), and (C) Molecular weight of fluorescence-labelled polymer particles (GPC). Error bars indicate the margin of error attributed to the error in molecular weight determination by GPC (±10%).

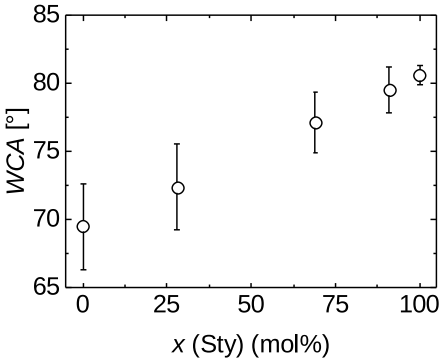

Figure S 2. Water contact angle (WCA) of hot-press molded polymer discs of the (co)polymer library.

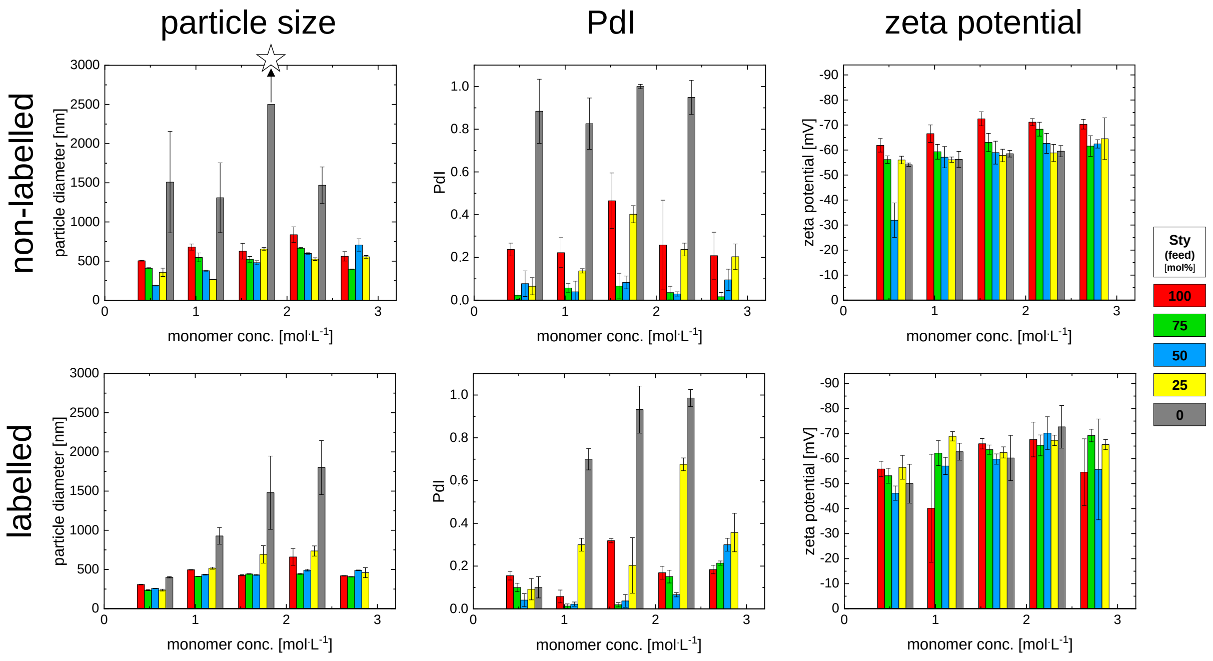

Figure S 3. Particle sizes and polydispersity indices (PdI) from DLS analysis as well as zeta potential, all for dialyzed particle suspensions, depending on the feed ratio of styrene (Sty) and the comonomer concentration. Error bars indicate the standard deviation of the measurements; the asterisk at the diagram for particle size of non-labelled particles indicates large aggregates above 5000 nm.

2.1. Structure-properties relationships in particle library

A particle formation model is proposed with the simplifications (i) of a direct proportionality of the zeta potential and the charge density of the particle, (ii) that only a single charged moiety per polymer molecule is present, and (iii) the density of the particles is homogenous, a facile relationship between the particle size *d*, the zeta potential *z*, and the molecular weight of the polymer chains $\overline{M}_n$ was found: According to the Gouy-Chapman theory and the Grahame equation, respectively, there is a direct relationship between the surface charge density and the surface potential, respectively, as expressed by Supp. Eq. 1 [5], where σ_{part} is the charge density of the particles, *c* the ion concentration, N_A Avogadro's constant, ε_r and ε_0 the dielectric permittivity of the medium and vacuum, respectively, k_B the Boltzmann constant, *T* the temperature, *e* the elementary charge, and *z* the zeta potential of the particles. For low *z*, a direct proportionality of the potential and the charge density of the particle can be assumed (Supp. Eq. 2) [5]. Furthermore, the specific charge of a polymer chain q_{poly} is indirectly proportional to the molecular weight of the polymer, hypothesizing that one charged moiety per polymer chain is introduced by the initiator (Supp. Eq. 3) as also assumed in other studies [6]. When assuming that all charged groups are located at the surface and no other charged groups are present, than will (i) σ_{part} be directly proportional to the number of polymer chains n_{poly} that a particle is composed of and (ii) q_{poly} be indirectly proportional to the surface area A_{part} of the particle (Supp. Eq. 4). The number of polymer chains n_{poly} per particle can be estimated by the ratio of the particle volume V_{part} and the volume of the polymer chain V_{poly}, which is for its part directly proportional to $\overline{M}_n$ (Supp. Eq. 5), hypothesizing a homogenous density of the particles. Assuming perfect spherical shape of the particles, the ratio between V_{part} and A_{part} is given by Supp. Eq. 6. Proportionality factors are denoted as k_1, k_2, k_3, and k_4.

$$\sigma_{part} = \sqrt{8cN_A\varepsilon_r\varepsilon_0 k_B T} \cdot \sinh\left(\frac{e \cdot z}{2k_B T}\right) \quad \text{(Supp. Eq. 1)}$$

$$z = k_1 \cdot \sigma_{part} \quad \text{(Supp. Eq. 2)}$$

$$q_{poly} = k_2 \cdot \frac{1}{M_n} \quad \text{(Supp. Eq. 3)}$$

$$\sigma_{part} = k_3 \cdot \frac{n_{poly} \cdot q_{poly}}{A_{poly}} \quad \text{(Supp. Eq. 4)}$$

$$n_{poly} = \frac{V_{part}}{V_{poly}} = \frac{V_{part}}{k_4 \cdot \overline{M_n}} \quad \text{(Supp. Eq. 5)}$$

$$\frac{V_{part}}{A_{part}} = \frac{d}{6} \quad \text{(Supp. Eq. 6)}$$

$$k = \frac{6 \cdot k_4}{k_1 k_2 k_3} \quad \text{(Supp. Eq. 7)}$$

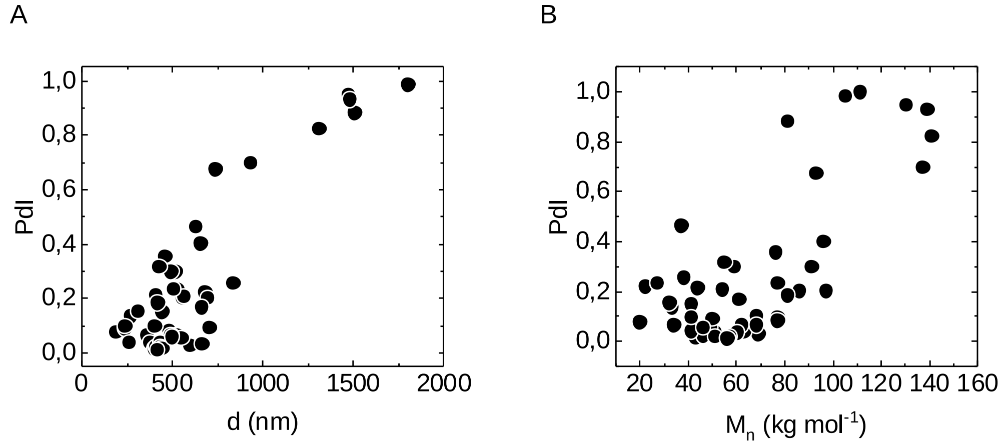

Figure S 4. Single-parameter correlation of the (co)polymer particle library: (A) particle diameter (d) vs polydispersity index (PdI) by DLS and (B) number average molecular weight (M_n by GPC) vs PdI (DLS).

2.2. Biocompatibility of Synthesized Particle Library

The presence of extractable bacterial lipopolysaccharides (LPS) was studied with the LAL-test, which demonstrated that all extracts were practically endotoxin free (<0.06 EU·mL^{-1}; data not shown). It is a specific feature of the HEK-Blue™-hTLR4 cells to get in direct contact with the particles and sense particle-bound LPS. This reporter cell assay can only be conclusive, when the material does not induce substantial cell death, which was the case for all members of the particle library (viability >60%). The cell test has a detection limit of 0.01 ng·mL^{-1} LPS, which corresponds to 0.05 EU·mL^{-1} [7]. As control cell lines, HEK-Blue™-Null2 cells without TLR4 expression were used. None of the particles induced a substantial activation of the HEK-Blue™-hTLR4 cells (Figure S 5), nor of the HEK-Blue™-Null2 cells (data not shown). These data indicate a low-germ manufacturing process of the particle library.

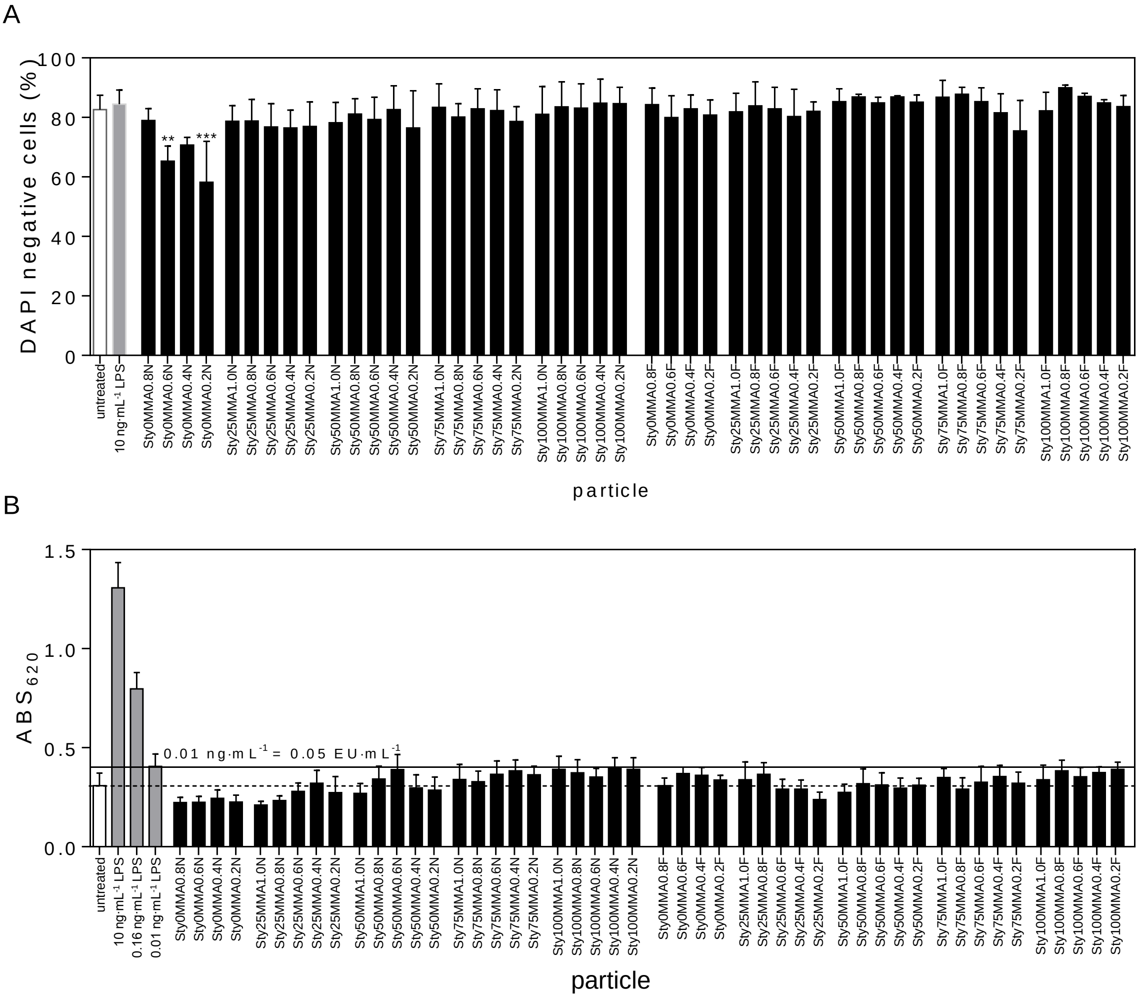

Figure S 5. Investigation of cell toxicity and absence of particle-bound endotoxins using HEK-Blue^{TM}-hTLR4 cells. (A) Cell survival (%) as determine by flow cytometry after DAPI staining. (B) Endotoxin determination in harvested cell culture supernatants via QuantiBlue^{TM} assay to measure the LPS-induced secretion of alkaline phosphatase. (n = 3 independent experiments; mean ± SD). Data were statistically compared with the untreated cells using the One-way-Anova test combined with the Dunnett's post hoc test (** for p<0.01, *** for p<0.001).

2.3. Gating Strategy for the Flow Cytometry and Uptake Studies

The uptake studies were performed with the fluorescence labelled particles. The cells were titrated with an increasing number of particles to observe characteristic uptake curves. Particle uptake was assessed by flow cytometry. The gating strategy comprised a scatter gate (SSC-A vs. FSC-A) to exclude cell debris, a single cell gate (FSC-H vs. FSC-A) to exclude cell aggregates, an eFluor670 gate (Fluorescein-A vs. eFluor670-A) to clearly distinguish between (labelled) cells and free particles, a live cell gate (DAPI-A vs. Fluorescein-A) to discriminate dead cells, and finally a particle-positive cell gate (Fluorescein-A vs. eFluor670-A). The scatter gate showed an increase in the side scatter in case of the particle treated cells compared to the negative control, which already was an indication for significant particle uptake

[8]. The live cell gate included the Fluorescein channel as it was found that particle fluorescence partially interfered with the DAPI channel. For this, the gate was adapted to avoid false-positive dead cell assessments (Figure S 6).

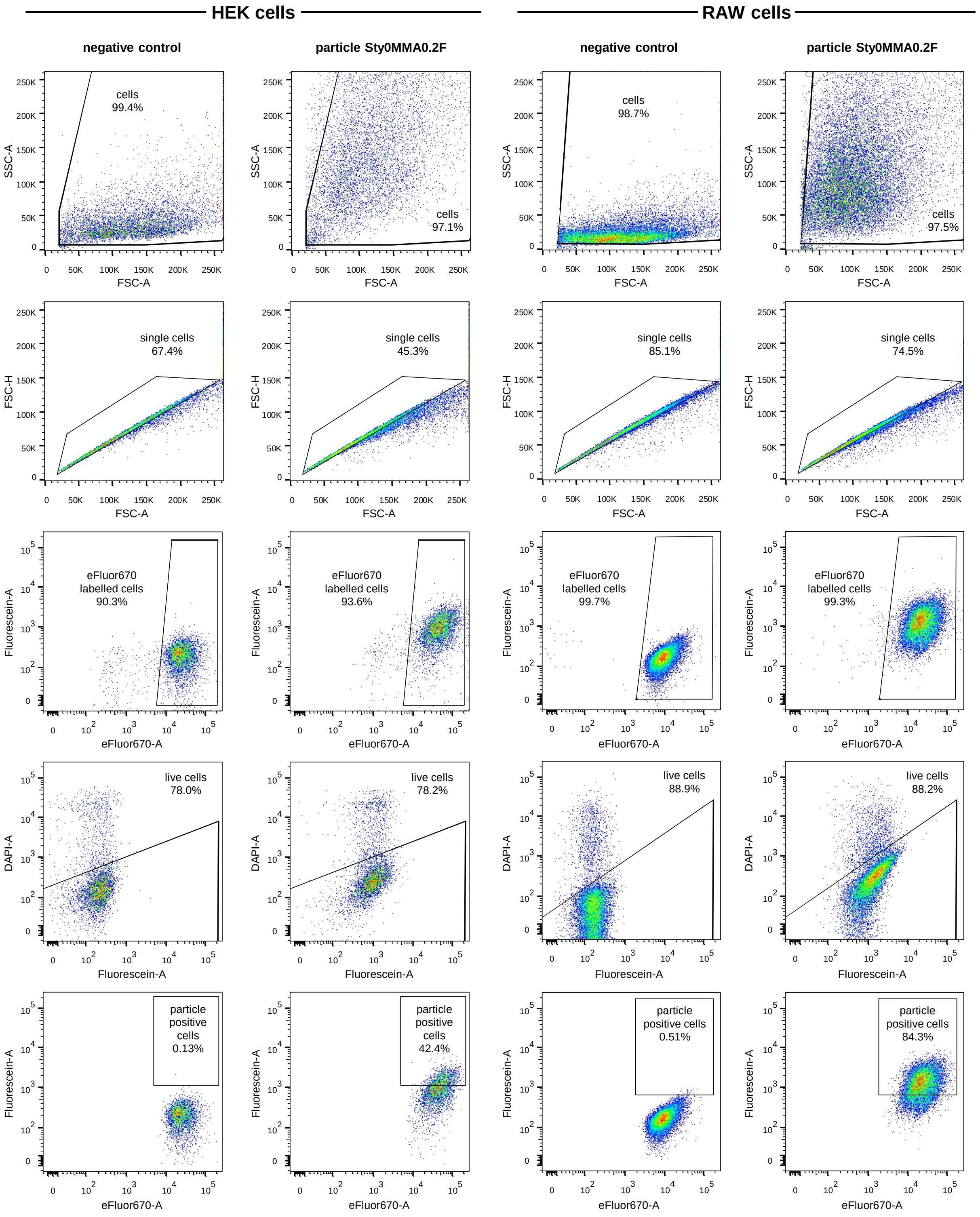

Figure S 6. Gating strategy for the flow cytometry based determination of particle uptake by HEK and RAW cells. Particles Sty0MMA0.2F (80 µg·mL^{-1}) have been taken as representative example. SSC: side scatter, FSC: forward scatter, A: area, H: height.

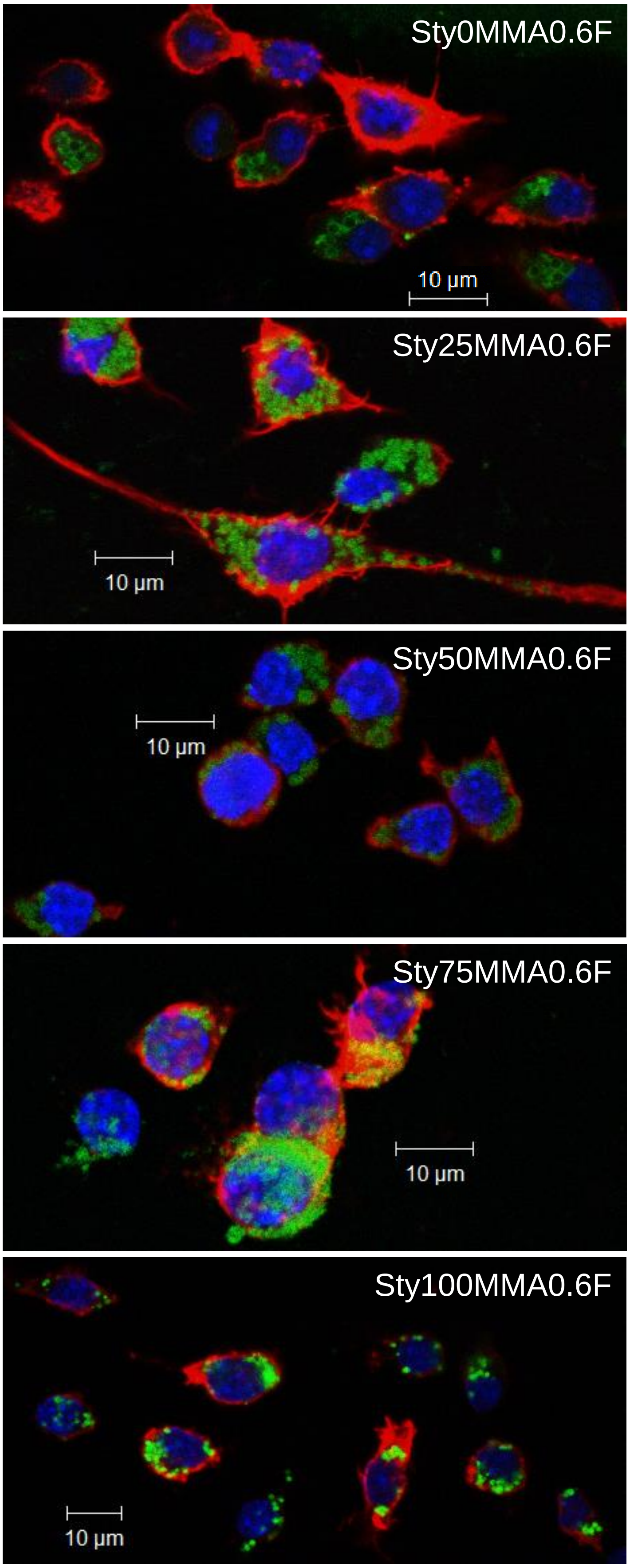

Figure S 7. Investigation of nanoparticle cell interaction. Confocal laser scanning microscopy analysis of RAW cells treated with particles ($5 \cdot 10^5$ cells suspended in 200 µL with 80 $\mu g \cdot mL^{-1}$ of particles). Exemplary images for selected compositions to illustrate the intracellular localization of particles (stained green with fluorescein during synthesis) as observed for all particle types. Cells were fixated and stained with Alexa- fluor 555 Phalloidin (actin, red fluorescence), and DAPI (nucleus, blue fluorescence).

3. References

[1] S. Baudis, A. Lendlein, M. Behl, High Throughput Characterization of Polymer Libraries by Diffuse Reflectance Infrared Spectroscopy, Macromol. Mater. Eng. 299(11) (2014) 1292-1297.

[2] S. Baudis, A. Lendlein, M. Behl, High-Throughput Synthesis as a Technology Platform for Copolymer Libraries, Macromol. Symp. 345(1) (2014) 105-111.

[3] F. Friess, T. Roch, B. Seifert, A. Lendlein, C. Wischke, Phagocytosis of spherical and ellipsoidal micronetwork colloids from crosslinked poly(ε-caprolactone), International Journal of Pharmaceutics 567 (2019) 118461.

[4] C. Wischke, S. Mathew, T. Roch, M. Frentsch, A. Lendlein, Potential of NOD receptor ligands as immunomodulators in particulate vaccine carriers, Journal of controlled release : official journal of the Controlled Release Society 164(3) (2012) 299-306.

[5] F.L. Marten, Vinyl alcohol polymers, in: H.F. Mark (Ed.) Encyclopedia of Polymer Science and Technology (4th Edition), John Wiley & Sons, Inc., New York, 2014, pp. 686-724.

[6] T.R. Aslamazova, K. Tauer, On the colloidal stability of poly(methyl methacrylate) and polystyrene particles prepared with surface-active initiators, Colloids and Surfaces A: Physicochemical and Engineering Aspects 300(3) (2007) 260-267.

[7] M.B. Gorbet, M.V. Sefton, Endotoxin: the uninvited guest, Biomaterials 26(34) (2005) 6811-7.

[8] A. Cossarizza, H.-D. Chang, A. Radbruch, S. Abrignani, R. Addo, M. Akdis, I. Andrä, F. Andreata, F. Annunziato, E. Arranz, P. Bacher, S. Bari, V. Barnaba, J. Barros-Martins, D. Baumjohann, C.G. Beccaria, D. Bernardo, D.A. Boardman, J. Borger, C. Böttcher, L. Brockmann, M. Burns, D.H. Busch, G. Cameron, I. Cammarata, A. Cassotta, Y. Chang, F.G. Chirdo, E. Christakou, L. Čičin-Šain, L. Cook, A.J. Corbett, R. Cornelis, L. Cosmi, M.S. Davey, S. De Biasi, G. De Simone, G. del Zotto, M. Delacher, F. Di Rosa, J. Di Santo, A. Diefenbach, J. Dong, T. Dörner, R.J. Dress, C.-A. Dutertre, S.B.G. Eckle, P. Eede, M. Evrard, C.S. Falk, M. Feuerer, S. Fillatreau, A. Fiz-Lopez, M. Follo, G.A. Foulds, J. Fröbel, N. Gagliani, G. Galletti, A. Gangaev, N. Garbi, J.A. Garrote, J. Geginat, N.A. Gherardin, L. Gibellini, F. Ginhoux, D.I. Godfrey, P. Gruarin, C. Haftmann, L. Hansmann, C.M. Harpur, A.C. Hayday, G. Heine, D.C. Hernández, M. Herrmann, O. Hoelsken, Q. Huang, S. Huber, J.E. Huber, J. Huehn, M. Hundemer, W.Y.K. Hwang, M. Iannacone, S.M. Ivison, H.-M. Jäck, P.K. Jani, B. Keller, N. Kessler, S. Ketelaars, L. Knop, J. Knopf, H.-F. Koay, K. Kobow, K. Kriegsmann, H. Kristyanto, A. Krueger, J.F. Kuehne, H. Kunze-Schumacher, P. Kvistborg, I. Kwok, D. Latorre, D. Lenz, M.K. Levings, A.C. Lino, F. Liotta, H.M. Long, E. Lugli, K.N. MacDonald, L. Maggi, M.K. Maini, F. Mair, C. Manta, R.A. Manz, M.-F. Mashreghi, A. Mazzoni, J. McCluskey, H.E. Mei, F. Melchers, S. Melzer, D. Mielenz, L. Monin, L. Moretta, G. Multhoff, L.E. Muñoz, M. Muñoz-Ruiz, F. Muscate, A. Natalini, K. Neumann, L.G. Ng, A. Niedobitek, J. Niemz, L.N. Almeida, S. Notarbartolo, L. Ostendorf, L.J. Pallett, A.A. Patel, G.I. Percin, G. Peruzzi, M. Pinti, A.G. Pockley, K. Pracht, I. Prinz, I. Pujol-Autonell, N. Pulvirenti, L. Quatrini, K.M. Quinn, H. Radbruch, H. Rhys, M.B. Rodrigo, C. Romagnani, C. Saggau, S. Sakaguchi, F. Sallusto, L. Sanderink, I. Sandrock, C. Schauer, A. Scheffold, H.U. Scherer, M. Schiemann, F.A. Schildberg, K. Schober, J. Schoen, W. Schuh, T. Schüler, A.R. Schulz, S. Schulz, J. Schulze, S. Simonetti, J. Singh, K.M. Sitnik, R. Stark, S. Starossom, C. Stehle, F. Szelinski, L. Tan, A. Tarnok, J. Tornack, T.I.M. Tree, J.J.P. van Beek, W. van de Veen, K. van Gisbergen, C. Vasco, N.A. Verheyden, A. von Borstel, K.A. Ward-Hartstonge, K. Warnatz, C. Waskow, A. Wiedemann, A. Wilharm, J. Wing, O. Wirz, J. Wittner, J.H.M. Yang, J. Yang, Guidelines for the use of flow cytometry and cell sorting in immunological studies (third edition), European Journal of Immunology 51(12) (2021) 2708-3145.
